# A Perspective on Unintentional Fragments and Their Impact on the Dark Metabolome, Untargeted Profiling, Molecular Networking, Public Data, and Repository Scale Analysis

**DOI:** 10.1021/jacsau.5c01063

**Published:** 2025-12-01

**Authors:** Yasin El Abiead, Ipsita Mohanty, Shipei Xing, Adriano Rutz, Vincent Charron-Lamoureux, Tito Damiani, Wenyun Lu, Gary J. Patti, Nicola Zamboni, Oscar Yanes, Pieter C. Dorrestein

**Affiliations:** † Skaggs School of Pharmacy and Pharmaceutical Sciences, 8784 University of California San Diego, 9500 Gilman Drive, San Diego, California 92093-0751, United States; ‡ Institute for Molecular Systems Biology, 27219 ETH Zurich, Otto-Stern-Weg 3, Zurich 8093, Switzerland; § Department of Biochemistry of Plant Specialized Metabolites, 89220 Institute of Organic Chemistry and Biochemistry of the Czech Academy of Sciences, Flemingovo Náměstí 542/2, Prague 160 00, Czech Republic; ∥ Lewis Sigler Institute for Integrative Genomics and Department of Chemistry, 6740 Princeton University, Princeton, New Jersey 08544, United States; ⊥ Department of Chemistry, Genetics, and Medicine, and Center for Mass Spectrometry and Metabolic Tracing, Washington University, St. Louis, Missouri 63110, United States; # Department of Electronic Engineering & IISPV, Universitat Rovira i Virgili, Tarragona 43007, Spain; ¶ CIBER de Diabetes y Enfermedades Metabólicas Asociadas (CIBERDEM), Instituto de Salud Carlos III, Madrid 28029, Spain; ∇ Collaborative Mass Spectrometry Innovation Center, Skaggs School of Pharmacy and Pharmaceutical Sciences, University of California San Diego, La Jolla, California 92093, United States; ○ Center for Microbiome Innovation, University of California San Diego, La Jolla, California 92093, United States

**Keywords:** metabolomics, mass spectrometry, dark metabolome, electrospray ionization, in-source fragmentation, analytical artifact

## Abstract

In/postsource fragments (ISFs) arise during electrospray ionization or ion transfer in mass spectrometry when molecular bonds break, generating ions that can complicate data interpretation. Although ISFs have been recognized for decades, their contribution to untargeted metabolomics—particularly in the context of the so-called “dark matter” (unannotated MS or MS/MS spectra) and the “dark metabolome” (unannotated molecules)—remains unsettled. This ongoing debate reflects a central tension: while some caution against overinterpreting unidentified signals lacking biological evidence, others argue that dismissing them too quickly risks overlooking genuine molecular discoveries. These discussions also raise a deeper question: what exactly should be considered part of the metabolome? As metabolomics advances toward large-scale data mining and high-throughput computational analysis, resolving these conceptual and methodological ambiguities has become essential. In this perspective, we propose a refined definition of the “dark metabolome” and present a systematic overview of ISFs and related ion forms, including adducts and multimers. We examine their impact on metabolite annotation, experimental design, statistical analysis, computational workflows, and repository-scale data mining. Finally, we provide practical recommendations—including a set of dos and do nots for researchers and reviewers—and discuss the broader implications of ISFs for how the field explores unknown molecular space. By embracing a more nuanced understanding of ISFs, metabolomics can achieve greater rigor, reduce misinterpretation, and unlock new opportunities for discovery.

## Graphical abstract

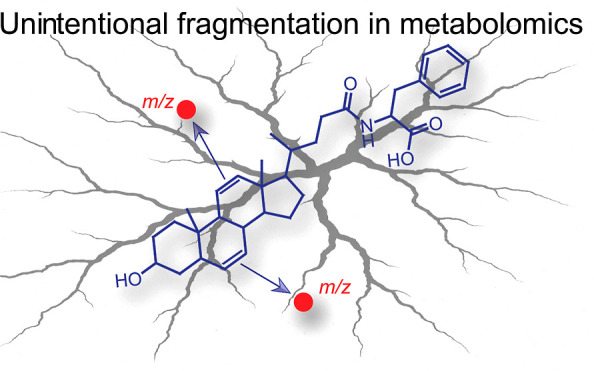

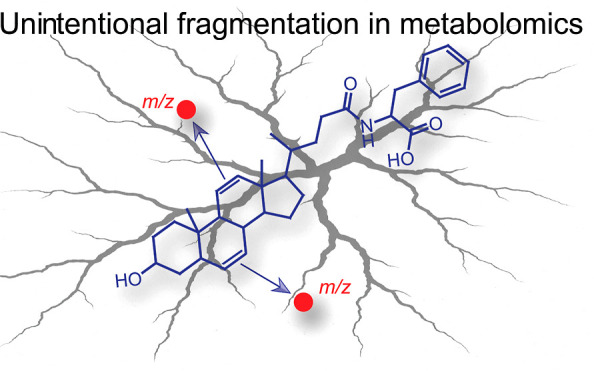

## Introduction

We were invited to write this perspective in response to an ongoing scientific discussion^1–10^ about the role of in-source fragments (ISFs) in untargeted LC–MS and LC–MS/MS based metabolomics, and the implications for the scale and nature of the dark matter/metabolome.^11–14^ We view this as an opportunity to clarify what is already known, highlight points of controversy, identify areas that merit deeper investigation, and propose how ISF-related data can be more effectively leveraged. It is important to note that at least some of the fragment ions typically attributed to in-source fragmentation (ISF)—fragment ions detected on the MS1 level, where generally no fragmentation is intended—may, in fact, arise from postsource fragmentation (Figure 1a). This distinction is grounded in the physical reality that ions within the mass spectrometer are routed, trapped, and accelerated by strong electrical fields. The origin of unwanted fragmentation can be investigated under special circumstances, but traditional LC–MS/MS experiments do not allow us to distinguish between these two possibilities. For the purposes of this perspective, we use the term “ISF” with the understanding that some of these ions may result from postsource processes. Our goal is to demonstrate how signals related to ISFs and other ion forms can be more effectively detected, interpreted, and integrated into data analysis pipelines. In particular, we discuss how these phenomena influence data interpretation—where evidence currently allows—and the implications for emerging computational approaches that compare millions of MS/MS—also called MS^2^—spectra. As with any perspective, this effort reflects the interpretations and reasoning of the authors, and we encourage readers to consider the broader range of viewpoints and supporting literature referenced throughout.

**Figure 1:**
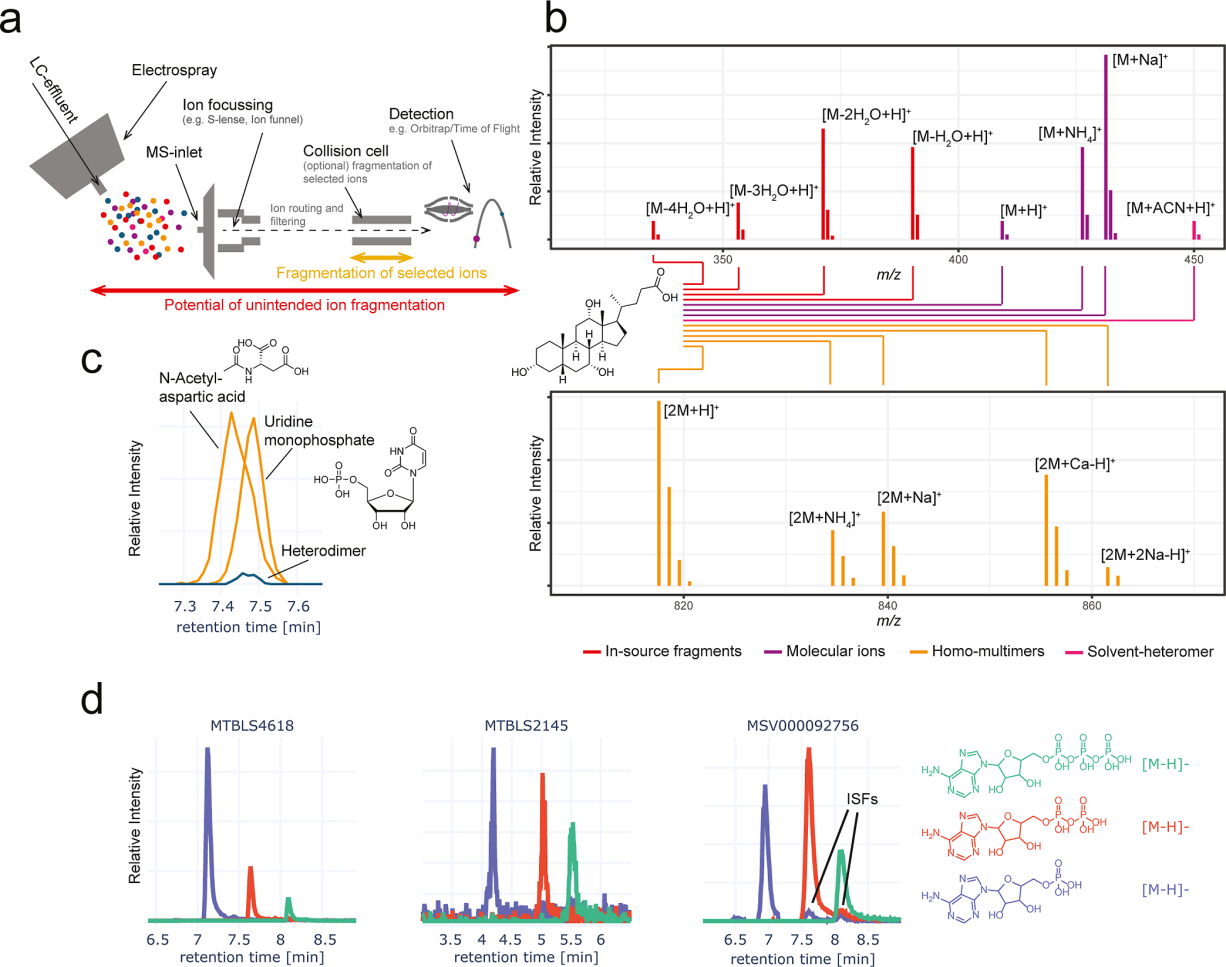
One molecule, many ions. (a) Schematic view of a LC outlet and source where in-source fragments and other ions are generated. Some of the ions that are ultimately detected could also be generated within the instrument postsource ion optics. (b) Possible ion forms that might be observed (adducts, including solvent adducts, dimers, including heterodimers, and ISFs, all with isotopes). This is showcased with the bile acid, cholic acid. (c) An experimental example of a heterodimer from MSV000089018, that can occur when two molecules partially coelute and thus co-ionize. Such ion species have been shown to generally account for less than 5% of biological features in metabolomics data sets.^16^ (d) It is well-known that during ESI ATP can fragment to ADP and AMP, and ADP into AMP.^19^ Without additional experiments, these ISFs can be recognized only through chromatographic separation as ISFs will coelute with their precursor ions. Here we show that these ISFs are not always observable across three different data sets (MSV000092756 [Q Exactive Plus], MTBLS2145 [impact II UHR-TOF],^20^ and MTBLS4618 [TripleTOF 6600]). Only in one data set are ISFs clearly visible at the MS1 level consistent with the notion that ISF detection is very much experiment dependent. Moreover, it should be noted that while coelution can be used to identify ISFs here this is not as trivial when the precursors are not known like here. Direct access to plots linking to the underlying raw data are provided in the associated links.

Despite ongoing debate around the relevance of ISFs in metabolomics, there is substantial common ground. There is broad consensus that not all features in untargeted metabolomics represent unique metabolites.^15^ For a molecule to be detected by LC–MS/MS, it must be ionized. While de- and protonated forms are commonly observed with acids and bases, a single metabolite can give rise to multiple ion forms. They include ISFs, adducts (e.g., NH_4_^+^, K^+^, Na^+^, Ca^2+^, Cl^–^ and formiate), and multimers, both homo- and hetero-multimers originating between different molecules (Figure 1a–c).^16–18^ The intensity of ion forms, including ISFs varies per compound and experiment (see example of ATP, ADP and AMP, where some display ISFs while others do not, Figure 1d). A single molecule can generate dozens, or in extreme cases even over 100 distinct ion signals, with or without ISFs, depending on the experimental context, analyte concentration, and compound class.^17^

While the community agrees on the importance of accounting for such forms in data interpretation, disagreement arises around the implications—whether one is examining a single LC–MS/MS file, an entire study, public repositories, or broader efforts in molecular discovery. *Much of the debate, then, reflects differences in scope and framing—essentially comparing apples to oranges*. However, we believe that if all parties in this discussion were to analyze the same data together, there would be strong consensus on how to validate annotations and design follow up studies to ensure reliability. The core divergence lies not in the facts, but in how those facts are interpreted, communicated and shaped opinions.

The significance of this debate extends well beyond a technical disagreement in data interpretation: it raises foundational questions about the future direction of discovery metabolomics. To nonexperts, the recent discussion risks creating the inaccurate perception that untargeted mass spectrometry data are largely artifacts or junk, casting doubt on its utility and, by extension, its place in multiomics strategies.^8^ This narrative can inadvertently delegitimize the collection and integration of metabolomics data in systems biology, suggesting it is too unreliable to contribute meaningfully. But beyond this perception issue, the core debate is a deeper challenge to our collective understanding of the metabolome itself.^1,2,11,14^

The debate directly influences how we define the goals, methods, and value proposition of metabolomics research. If we adopt a narrower view—that the majority of unannotated features are artifacts like ISFs representing already known molecules—then the urgency for discovering new molecular entities may diminish. Under this lens, the case for investing in large-scale discovery metabolomics weakens, effectively disincentivizing untargeted and unbiased analysis. However, if we instead recognize the metabolome as encompassing a vast and biologically meaningful chemical space—one that remains largely uncharted—then the imperative for continued innovation of annotation tools, and exploratory data analysis becomes even stronger. It justifies not only sustained, but expanded, support for method development, funding programs, and training of scientists equipped to explore this frontier. This moment mirrors a similar turning point from a decade ago in the natural products field, when researchers debated the untapped potential of small molecule discovery.^21–27^ Those conversations are fundamentally reshaping how the field prioritized biosynthetic diversity and dereplication strategies. Likewise, today’s metabolomics community stands at a crossroads—how we define and value the unknown will influence the trajectory of the discipline for years to come. Importantly, this debate is not unique to the science of small molecules.

The term *dark metabolome* has been used in a variety of ways across the literature.^11,13,28–32^ In some contexts, it refers specifically to the set of molecular features detected by LC–MS or LC–MS/MS—such as MS1 ion peaks with associated retention times, or MS/MS spectra—that remain unannotated, e.g. structurally unidentified. In others, it is used more broadly to describe the uncharacterized portion of the metabolome, including undetected molecules, present in a biological sample, or even beyond a single experiment—the totality of metabolite space yet to be discovered within a species. Depending on the definition adopted, estimates of the size of the dark metabolome can vary substantially. These estimates are influenced by several factors, including the performance of annotation strategies (i.e., the more features we can annotate, the smaller the dark metabolome appears), the analytical methodologies employed (such as serial extraction, orthogonal chromatographic approaches, or multiple ionization techniques to enhance chemical coverage), and the number of biological specimens analyzed (since interindividual variability increases observed molecular diversity). In the absence of a community-wide consensus, it is unsurprising that multiple—and sometimes conflicting—interpretations of the term persist.^14,33^

We argue that a clear and standardized definition of the *dark metabolome* and related terms is needed. Figure 2a provides a contextual framing of the diverse perspectives on the term. Interpretations range from the entirety of molecules present in organisms in their environmental context to the sample molecules accessible in a given metabolomics experiment. The *dark matter* of metabolomics (Figure 2b) describes the entirety of unidentified signals in a set of metabolomics raw data. Naturally, the term is highly related to the dark metabolome as the number of observed signals is often intuitively related to the size of the dark metabolome. However, such conclusions require nuance. As shown in Figure 2c, the way a signal is defined can vastly change downstream interpretations. Caution must be taken to not overestimate counts or diminish the potential for discovery. The weighting of this trade-off depends on the scientific question.

**Figure 2:**
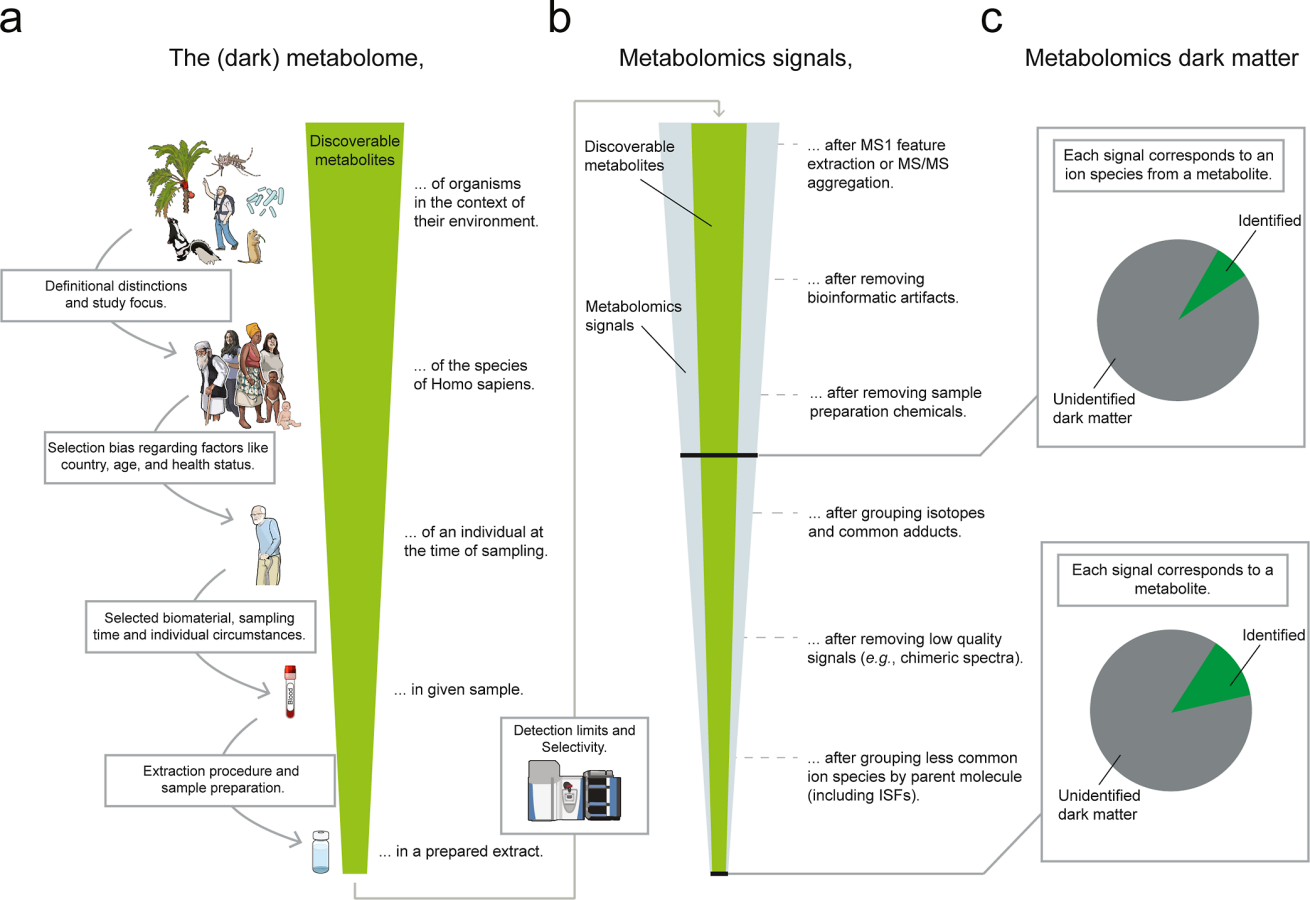
Definitional flavors of the (dark) metabolome and dark matter of metabolomics. (a) The size of the *dark metabolome* depends on the chosen reference point, which varies with the scientific question. (b) Metabolomics data processing can be performed on the raw data file derived from a single sample, a data set containing multiple samples or a whole repository. Unique ion species can be aggregated as unique MS/MS scans^34^ or MS1 based feature extraction.^35^ What constitutes a signal is context-dependent (e.g., an MS/MS spectrum, a consensus spectrum, or an LC–MS peak). Signals can be filtered or grouped to remove bioinformatic artifacts,^36,37^ uninterpretable features (such as chimeric spectra or ambiguous peaks),^38,39^ and redundant ion species. However, overly strict filtering may also diminish the potential for novel discovery.^40^ The order of the workflow steps can vary depending on preference and scientific question. (c) The *dark matter of metabolomics* refers to the portion of metabolomics signals that can not be identified. The meaning of this concept in the context of the *dark metabolome* depends on analyzed samples, the metabolomics signal processing steps made, and strategies used for annotation of the observed extracted signals.

Here, we define the dark metabolome of a given biological or environmental sample as comprising both nondetected and detected-but-unannotated compounds. Nondetected compounds arise from methodological and analytical limitations, including the type of chromatography (e.g., hydrophilic interaction liquid chromatography (HILIC) vs reversed phase chromatography (RPC)), extraction efficiency, ionization technique (e.g., ESI vs EI), the sensitivity of the mass spectrometer, and the overall selectivity of the methodology. This space of nondetected compounds includes both known chemical structures and truly novel, previously undescribed molecules. We define the dark matter of a metabolomics experiment, on the other hand, as the number of detected-but-unannotated features or MS/MS spectra within a specific data set. While the size of the dark matter in a data set can reflect part of the dark metabolome, the two are not equivalent. That is, the absence of detected-but-unannotated compounds in a single experiment does not imply the absence of a dark metabolome—it simply reflects the limitations of that particular analysis.

Thus, far, much of the main discussion has focused on the analytical details associated with the portion of the metabolome that is detectable by mass spectrometry yet remains unannotated—the *dark matter of metabolomics*. These are the *m*/*z* features that cannot be structurally identified using MS or MS/MS data. Such features may originate from known compounds or from previously undescribed molecules. At this point, it is also important to reemphasize something that should have broad consensus: not all molecules detected by LC–MS or MS/MS in a biological sample are metabolites, but all metabolites are, by definition, molecules. While this may seem obvious, several factors complicate their annotation and classification. In MS1, challenges include the formation of adducts, ISF, and the presence of background ions—exogenous contaminants from solvents, plasticware, or instrumentation—that without careful interpretation, may be mistakenly interpreted as endogenous metabolites. In MS/MS, structural annotation—the process of narrowing down plausible molecular identities—is hindered by multiple limitations: insufficient availability and coverage of reference libraries, the inclusion (or lack thereof) of matching precursor ion forms (adducts) found in the experiment, inconsistencies in experimental conditions between reference and query spectra, and the overall quality of reference spectra. Many spectra contain few fragment ions, exhibit low signal-to-noise ratios, or are chimeric—containing fragments from multiple coisolated precursor ions—making it difficult for computational tools to confidently assign structures. Additionally, the performance of annotation algorithms can be affected by suboptimal scoring metrics and inconsistencies across spectral databases. Collectively, these technical and analytical aspects affect our ability to assign structures to a large fraction of detectable ion forms and thereby constrain efforts to define, characterize, and ultimately reduce the so-called “dark metabolome”.

The current discourse around “dark metabolome” and ISFs and other experimental artifacts in metabolomics parallels longstanding challenges in microbiome science. There, researchers have long struggled with how to interpret sequencing data that fall outside of reference databases.^41^ Consider titles such as *“Most Microbial Species Are ‘Dark Matter’”*,^42^ *“Microbial dark matter could add uncertainties to metagenomic trait estimations”*,^43^ *“Running after ghosts: are dead bacteria the dark matter of the human gut microbiota?”* ^44^ and *“The bright side of microbial dark matter: lessons learned from the uncultivated majority”*.^45^ These papers and perspectives underscore how deeply rooted the tension between differing viewpoints is. In the case of the dark microbiome, the core of the viewpoints centers on this divide: some caution against overinterpreting unknown sequences without clear biological evidence, while others argue that prematurely discarding them risks overlooking novel biology and stifling discovery.

Both metabolomics and microbiome research have grappled with definitional ambiguity—specifically, what qualifies as “dark”? Does it refer to the unknown, the unculturable, the unquantifiable, the artifactual, or the analytically unreliable? This semantic uncertainty has led to confusion and diverging research priorities. We believe the issue lies in the term “*dark*” itself: it lacks precision and may unintentionally overstate the significance of unannotated data, suggesting mystery where there may simply be technical limitations.

One potential framework for further navigating this ambiguity associated with the dark metabolome is the Rumsfeld matrix, which categorizes knowledge into *known knowns*, *unknown knowns*, *known unknowns*, and *unknown unknowns*. The quadrant defines what molecules are annotated and expected to be present, what is detected but cannot yet be annotated, molecules that should be present but are not annotated and presence of molecules that have not been described before. Applying this framework to metabolomics raises new questions that are also a challenge in the context of the term dark metabolome: Should we use it at the level of MS1 features, MS/MS spectra, or only for fully resolved molecular structures? While appealing, such classifications also risk oversimplification without clearer definitions and shared community standards.

Yet, one researcher’s discarded noise data may be another’s discovery. Microbiome science has shown us that signals once discarded as sequencing artifacts were, with better algorithms, improved instrumentation, and greater contextual understanding, ultimately recognized as meaningful—driving major discoveries in microbial and viral diversity, gene function, and host–microbe interactions.^46–49^ Metabolomics now stands at a similar inflection point. How we choose to define, classify, and value molecular features that are without an assignment of a structure will shape not only how we allocate resources and train the next generation of scientists, but also how we uncover new biology relevant to human, environmental, and planetary health.

The most recent scientific discussion about ISFs was sparked by a paper entitled “*The hidden impact of in-source fragmentation in metabolic and chemical mass spectrometry data interpretation*”.^1^ The paper’s central argument is based on a striking numerical mismatch: the human genome encodes approximately 20,000 genes, only a subset of which are enzymes, yet untargeted LC–MS and LC–MS/MS analyses routinely detect far more molecular features that are obtained from detectable ions formed from metabolites. From this discrepancy, the authors argue that many of these features are unlikely to represent distinct endogenous metabolites and may instead be analytical artifacts—particularly ISFs. Supporting this hypothesis, the study analyzed approximately 931,000 known molecules and found that around 70% of ions detected under nominally 0 V collision-induced dissociation (CID) conditions could be attributed to ISFs. The high proportion of such fragments led the authors to conclude that a substantial number of unknown signals in metabolomics data sets may in fact represent misassigned fragments rather than novel metabolites. Consequently, the study suggests that ISFs—and postsource fragmentation more specifically—may have a much greater and previously underappreciated impact on data interpretation, implying that the true extent of the dark metabolome may be smaller than previously believed.

The publication catalyzed significant discussion, not just within scientific publications but also across social media, blogs, and popular and respected science outlets such as Science and the Analytical Scientist. Eye-catching headlines such as “Phantom Metabolites”^50^ and “The Dark Metabolome: A Figment of Our Fragmentation?”^4^ amplified the narrative that up to 70% of metabolomics data may be artificial due to in-source fragmentation, potentially undermining the reliability of data interpretation. However, several scientists, including authors of this perspective, have offered alternative interpretations, questioning both the media narrative and the original study’s central claim regarding the hidden impact of ISFs on metabolomics data analysis.

First, it is important to clarify that ISFs/postsource fragments are a well-documented phenomenon of the ionization process.^19,51,52^ The metabolomics community has long recognized the potential for ISFs to lead to misannotations, particularly when fragment ions mimic the masses of known metabolites. Although this issue is generally not discussed explicitly in publications, most researchers working with LC/MS-based metabolomics have encountered it during data analysis. Despite being underreported, the topic is not entirely absent from the literature—searching PubMed for “in-source fragmentation” and “metabolomics” returns over 250 studies. In some cases, it leads to considerable effort and wasted resources before the signal is ultimately recognized as an artifact—typically before publication. Despite these challenges, we found no published studies in which ISFs have been shown to lead to incorrect biological conclusions. That said, one recent study by Houriet et al. (2025) demonstrates this potential in the context of glycosylated plant metabolites.^53^ In that work, in-source redundant features were mistakenly treated as independent analytes, leading to annotation errors and an overestimation of sample complexity. Even so, none of the colleagues we consulted could recall a case where an ISF directly misled interpretation at the biological level. If such published examples do exist, we would be very interested in learning about them, as they could provide valuable insights for future research.

Second, despite its title, the correspondence by Giera et al. (2024) does not demonstrate that ISFs have a hidden impact on the interpretation of real-world metabolomics data. Instead, it presents an observation from a 0 V CID MS/MS experiment on 931 K chemical standards—a remarkable technical achievement—resulting in the observation that 70% of postsource decay ions (incorrectly referred to as fragments formed at the source in the paper) had the same ions observed in MS/MS spectra at higher CID voltages.^1,54^ Under these conditions, however, the instrument’s electronics differ from those in MS1-only mode, and no control was included to determine whether such differences contribute to postsource decay ions. Follow-up work has shown that ISF conditions without CID produce the same fragment ions as 0 V CID, but with differences in fragment ion intensities. In their selected examples presented, and under their instrument settings, ISF produced higher fragment ion intensities than 0 V CID.^54^ This outcome, however, warrants additional research as it is not expected to be universal as ISFs depend strongly on ionization and instrument parameters.

In addition, the biological relevance of the 0 V finding is limited as the experiment is conducted under nonbiological conditions: extremely high analyte concentrations and low chemical complexity, which are rarely encountered in biological extracts. Such experimental conditions differ significantly from those found in untargeted metabolomics studies.^2,3^ Extrapolating findings from this *N* = 1 study to all metabolomics workflows is like claiming that because one apple tree has 100 apples, an orange tree must also have 100 fruits. In practice, ISF formation is context-dependent, shaped by a range of factors including instrument design, source settings, analyte concentrations, and compound class.^52,55–58^ These variables must be considered when assessing the potential impact of ISFs on data interpretation.

The third aspect of the counterpoint is this: a crucial step in discussions about unknown molecules in untargeted metabolomics is recognizing that the detection of a feature in MS1—whether it is an ISF, adduct, or other ion form—does not, in itself, indicate whether the underlying molecule is known or novel. Therefore, one cannot claim that ISFs increase or decrease the size of the dark metabolome—or question its existence—based solely on their presence. Any unannotated MS/MS spectrum may originate from either a known compound or a previously undescribed one. To accurately estimate the proportion of annotated versus unannotated molecules—the essence of the dark matter—one must first determine how many unique molecules are represented in the data set. This requires characterizing MS1 data, identifying ion clusters, and annotating their components. Only then can structural annotations from MS/MS spectra be correctly interpreted in relation to their MS1 origins.

We also question the logic by Giera et al. (2024) that the large number of unidentified spectra, which far exceeds the roughly 20,000 protein-coding genes in the human genome (only a fraction of which encode enzymes), can only be primarily attributed to technological artifacts. The number of human genes is, in fact, not the key determinant of metabolic complexity. Enzymes are inherently multifunctional and operate within modules and networks, enabling the production of a vast array of chemically diverse metabolites—lipids being an easy to understand example. Crucially, the primary source of metabolites is not the genome but the diet, with microbial transformations further expanding the chemical space observed in human samples. While we acknowledge that MS artifacts exist and contribute to data interpretation challenges, this interpretation oversimplifies the inherent biological and chemical diversity present in most metabolomes under study. For example, it is estimated that up to 90% of diseases involve exposures to molecules not encoded by the human genome. This includes dietary components and microbiome-derived metabolites.^59^ Molecules such as thiamine, tryptophan, or linoleic acid are not synthesized by human enzymes but are essential components of human metabolism, originating from diet or microbial activity. Moreover, the metabolome includes many biologically relevant compounds produced through nonenzymatic processes. These include oxidative stress markers such as certain prostaglandins (e.g., prostaglandin F_2_α) and leukotrienes (e.g., leukotriene B_4_), Maillard products observed in hyperglycemia, acylated glutathione conjugates that reduce toxicity, and nitrosothiols involved in redox signaling.^60–65^ To disregard these molecular species is to underestimate the true complexity of the metabolome and overstate the explanatory power of technological limitations alone.

While this perspective focuses primarily on the human metabolome, the challenges discussed apply broadly to LC–MS or LC–MS/MS data from both biological and nonbiological sources—including environmental samples from soil, oceans, rivers, and built habitats. Therefore, a broader and more inclusive definition of metabolome of any organism can be defined as the complete collection of small molecules present in a biological system at a given time. This includes:

- **Conserved metabolites** from core biochemical pathways (e.g., energy production, basic biosynthesis), which are shared across many organisms and represent evolutionarily ancient functions. This is dominated by primary metabolites.
- **Specialized metabolites** produced in specific tissues, organisms, or conditions, often serving roles in chemical defense, environmental adaptation, interorganism communication, or niche-specific survival strategies. This is dominated by secondary metabolites.
- **Exposome-derived compounds**, encompassing exogenous chemicals from diet, environment, pharmaceuticals, or microbiota. These may be transformed by metabolism, regardless of whether their processing steps are fully characterized (Figure 3).

**Figure 3:**
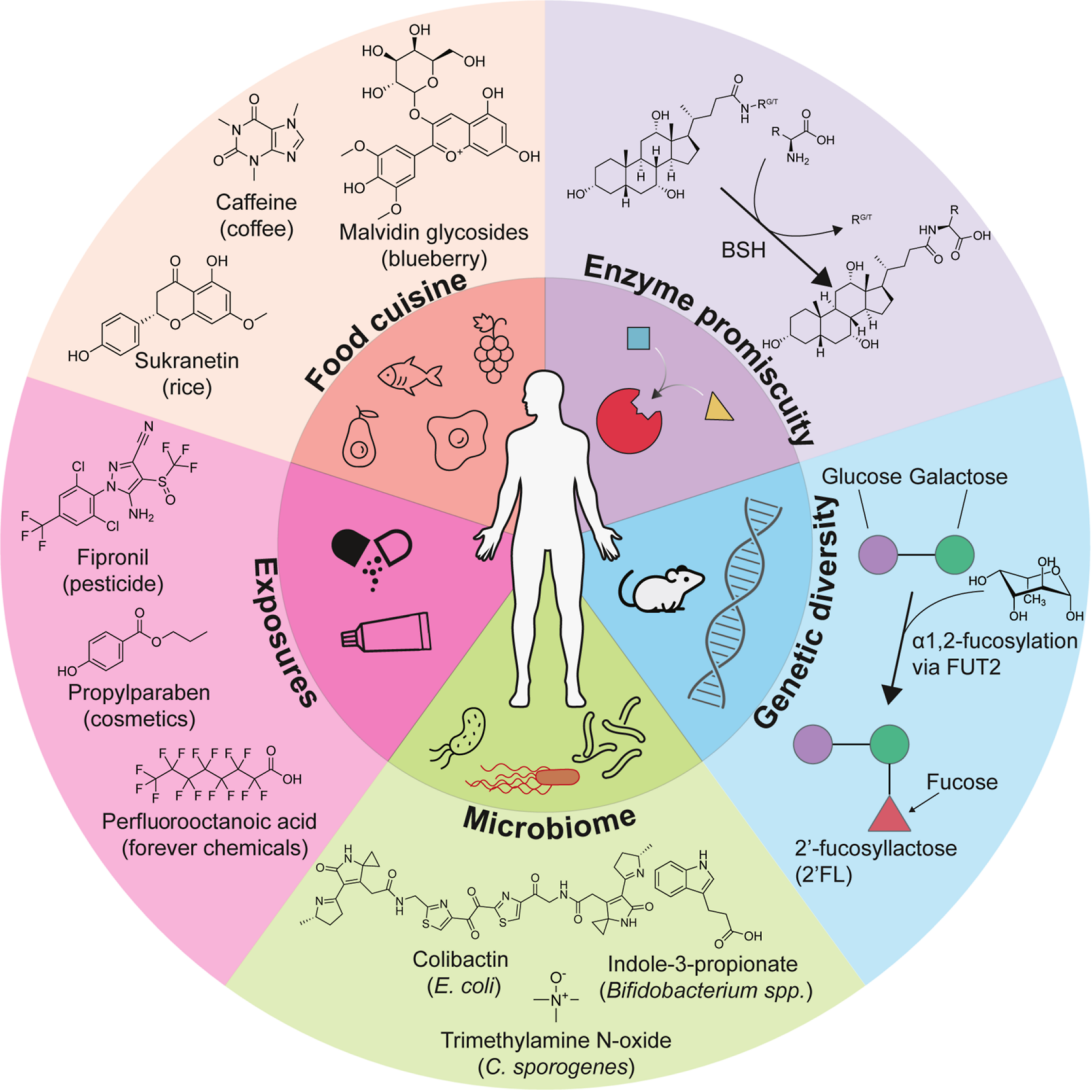
Sources contributing to the molecular diversity of the human metabolome. They are generally also relevant to other biological systems.

From our perspective, the only detectable ion forms in MS or MS/MS experiments that should be categorically excluded from any definition of the metabolome are those arising from nonbiological contaminants—specifically, compounds introduced through plasticware, solvents, or reagents, as well as those generated by artifactual reactions during sample preparation. These compounds lack biological relevance because they are not present in the biological system prior to sample handling and do not originate from endogenous metabolism, microbiota activity, or environmental exposures. In this context, therefore, it is essential to emphasize that detectability by mass spectrometry alone does not imply biological relevance or activity if they arise from the experiment rather than biology.^3,6^

Regardless of how one defines the boundaries of the metabolome, the metabolomics community remains divided. Some researchers argue that a vast, under-characterized “dark metabolome” still awaits discovery, while others are more skeptical of its scale. However, support for the former view continues to grow, reinforced by the steady pace of new discoveries. Even in extensively studied organisms such as *Escherichia coli*, mice, and humans, researchers routinely identify previously unreported metabolites—underscoring that current chemical databases are far from complete.^66–83^ Although precise numbers for the growth of human metabolites remain elusive, estimates from nonhuman systems suggest that approximately 1600 new small-molecule natural products (i.e., metabolites in those organisms) were reported annually between 1995 and 2015, a rate that has remained stable over time.^25^

Additional sources like Wikidata—a collaboratively curated knowledge base—can be used to trace the appearance of novel molecules over time. Although recent years are underrepresented due to curation delays (often >decade), it provides a useful long-term view of discovery trends. To reduce the effects of database curation delay bias, we focused on compounds reported between 1993 and 2013 and defined novelty based on InChIKey connectivity layers, which approximate the structural detail resolvable by mass spectrometry. During this 20 year window, the average annual number of newly reported molecular connectivities varied by taxon: plants contributed 3484, fungi 852, animals 546, and bacteria 438 per year. For specific model organisms, the total number of curated metabolites was lower: *Homo sapiens* (1099, mainly from Recon 2.2),^84^ *Arabidopsis thaliana* (650), *E. coli* (611, from models like iJO1366),^85^ and *Saccharomyces cerevisiae* (61). Hence, these reports represent a lower boundary of discovery over time.

Public resources also support discovery through large-scale spectral matching. Using panReDU^86^ filtering of human samples deposited in GNPS between 2014 and 2024, we estimate that an average of 761 new molecules per year were added to reference libraries with MS/MS matches to human data. Importantly, there is no indication that the pace of discovery is slowing—further supporting the idea of a substantial and still-growing dark metabolome. And yet, the size of the dark metabolome is largely influenced by sample diversity. Most of our knowledge comes from fasting-state samples common in clinical studies, yet a single LC–MS/MS run captures only a snapshot. Broader sampling—across time (such as circadian, seasonal cycles or lifetime), tissues, individuals, and conditions—reveals a far more dynamic metabolome, with variation further shaped by diet, microbiome, environment, and lifestyle. Encouragingly, innovations over the past decade—including the push toward public data sharing—are beginning to accelerate metabolite discovery. As this momentum continues, it is reasonable to anticipate that a majority of human-derived molecules will be structurally characterized within the coming decade(s).

In untargeted LC–MS/MS experiments, every MS/MS spectrum is initially part of dark matter—that is, unannotated spectral data. However, unannotated spectral data do not necessarily represent novel molecules. To move from unannotated spectra to novel molecules within a data set, one must first group ion features (such as adducts, in-source fragments, multimers and isotopes) into clusters that represent unique molecular entities. This distinction is essential because, when identical samples are analyzed across different laboratories using similar high-resolution MS instruments, feature overlap can be as low as 20–30%. These discrepancies are largely driven by differences in ion form detection—such as ISF, charge state distribution, and adduct formation—rather than by the presence of different underlying molecules.^87^ Only after proper ion grouping and annotation—via spectral library matching or alternative structure elucidation approaches—can one begin to assess how many molecular entities in a data set are annotated. Even then, the majority often remain unidentified. For example, in a reference NIST data set, over 82% of deconvoluted molecules lacked structural annotation, despite extensive data curation that included blank subtraction, filtering based on linear response in a dilution series, and removal of polymer-related features.^2^ These findings provide strong evidence for dark matter in metabolomics—that is, detectable but unannotated peaks—and also support the existence of a measurable dark metabolome in that sample.^88^

However, this 82% figure should not be generalized across all metabolomics experiments, as the proportion of unannotated molecules is highly dependent on sample type and experimental conditions. In fact, even the same data set can yield different annotation rates when processed using alternative computational workflows or parameter settings. Some data sets will have higher proportions of annotated metabolites and others will have less. In 2017, it was demonstrated on *E. coli* that stable-isotope labeling of organisms can be utilized to differentiate molecules of biological origin from any other molecule in a metabolomics data set.^89^ Later, in other stable-isotope labeling studies of simple model organisms, 67% (359/538) of *S. cerevisiae* metabolites and 52% (133/255) of *E. coli* metabolites remained unidentified even after ion form deconvolution,^90^ which was consistent with the study performed in 2017. In one of the most extensively studied human cell lines, 293T (i.e., not affected by diet intake, microbiome nor exposures), isotope labeling revealed that 87% (732/871) of labeled molecular entities could not be assigned to known compounds.^88,91,92^ This resulted in the discovery of >300 previously unknown biochemical reactions.

Annotation likelihood also varies with metabolite prevalence. In blood or plasma, if a molecular feature is detected in over 50% of samples, there is roughly a 50% chance it has a known annotation. Conversely, this means that even among the most consistently detected metabolites, half remain unannotated. And these frequently observed features represent only a small portion of the total metabolome. For low-abundance or less frequently detected molecules, annotation rates often fall well below 5%.^93^ Taken together, these data show that the size and scale of the dark metabolome is not a byproduct of in- and postsource fragmentation or misannotation. Rather, it reflects a genuine and biologically meaningful gap in our current knowledge.

The perspective by Giera et al. (2024) misses other major molecular inputs (Figure 3), such as diversity of diets and worldwide cuisines and therefore contributes to their large diversity of dietary ingredients and compounds. In addition there is a large array of environmental exposures. Diet and other exposures contribute many molecules, most of which undergo metabolism—including ones that are not generated by enzymes. That perspective also overlooks the genetic diversity and metabolic capabilities of the human-associated microbiome. Humans, host, bacteria,^94–102^ archaea,^103–105^ fungi,^106,107^ helminths, eukaryotic parasites,^108,109^ and there are even virus/phage-encoded enzymes^110,111^ —all of which can generate molecules that become part of a human’s metabolism. Finally one enzyme can take many substrates to generate multiple products—e.g. cytochromes P450 are well-known to metabolize a wide range of human made molecules such as drugs, pesticides and plasticizers.^112^

## How to Distinguish ISFs and Other Ion Forms?

There are, however, scenarios where distinguishing ISFs in data from biological samples could be helpful or even necessary. For example, when estimating the mass of an unknown compound or determining how many distinct molecules underlie a set of detected ion features, resolving ISFs—and other ion forms—becomes crucial.

Fortunately, a wide range of computational tools have been developed to facilitate this task (Figure 4). Starting over 15 years ago, early methods were implemented as standalone R scripts or embedded in automated data processing pipelines.^17,113–122^ These methods typically rely on retention time alignment, peak shape similarity, and intensity correlation (e.g., coelution mapping), and interpretation of characteristic *m*/*z* differences—which continue to be refined^3^ to detect coeluting ions likely derived from the same molecule (Figures 4a and S1a–d). Building on these foundations, correlation-aware network visualizations have further enabled the reconstruction of ISF clusters and provided insights into relationships between ions and their corresponding metabolites.^123^

**Figure 4:**
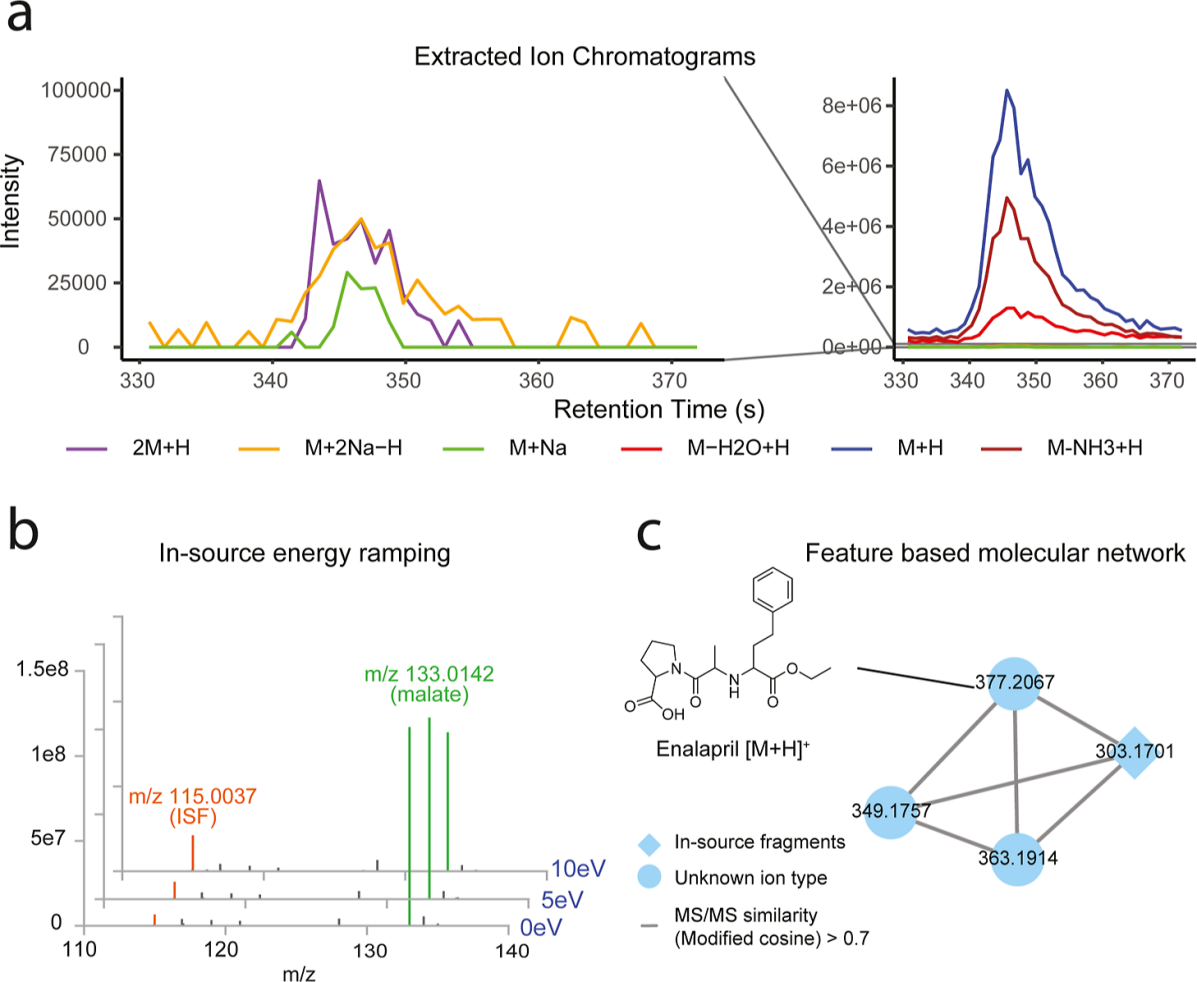
Strategies to group ISFs and other ion types: ISFs and adducts can be grouped in different ways as shown here for a selection of molecules. (a) It is possible to utilize peak shape correlation, which is more strict than general coelution. (b) An experimental approach to annotate ISF using in-source CID ramping: the signal intensity of the ISF will increase at 5 or 10 eV in-source CID compared to 0 eV, as demonstrated for the ISF at *m*/*z* 115.0037 from the metabolite malate (*m*/*z* 133.0142) in negative ion mode (raw data at https://massive.ucsd.edu/ with accession ID MSV000087131). For instruments without in-source CID function, alternative fragmentation techniques such as all ion fragmentation (AIF) can be utilized in a similar way. (c) Enalapril, an angiotensin-converting enzyme inhibitor, detected in two samples of the data set MSV000096589 is shown after feature based molecular networking in MZmine 4. Ion species with similar MS/MS, as determined by modified cosine, are connected. The *m*/*z* value of each ion species is given on the node.

More specialized tools such as CAMERA,^115^ RAMClust,^124^ MISA,^125^ and others enable finding ion forms by leveraging peak correlation analysis between precursor ions and suspected ISFs (Figure S1c). This approach is especially valuable given the potential resemblance between the MS/MS spectra of ISFs and those of intact metabolites (e.g., the amine loss fragment of ornithine produces a spectrum similar to that of proline (Figure S1d,e)). Despite these advances, explaining the structural transformations that produce specific ISFs remains an active area of research.^16,18^ Importantly, all of the aforementioned computational tools enable retrospective analysis of ion forms from any existing LC–MS/MS data set. In contrast, other approaches rely on dedicated experimental designs. One such strategy is *in-source energy ramping* (Figure 4b), which involves injecting the same sample multiple times while gradually increasing the in-source energy allowing identification of in-source fragments. Features that intensify with higher energy are classified as ISFs, while unchanged signals serve as the reference. This technique was first introduced by Wang et al. in 2019 and has since been applied to investigate ISFs and broader ion speciation phenomena.^52,90,126,127^

Another strategy for recognizing ISFs is molecular networking—a powerful visualization method that reveals structural relationships between MS/MS spectra based on spectral similarity (Figure 4c). In these networks, nodes represent precursor ions, and edges connect those with similar fragmentation patterns. This approach groups structurally related ions—such as analogs, adducts, and ISFs—which may appear as star-like clusters around a central node or as separate nodes if their spectra differ significantly.^128^ The detection of ISFs depends on MS/MS coverage and fragmentation conditions; overlaying retention time information can help associate different ion forms of the same compound.

Over the past decade, its utility has expanded beyond the original modified cosine score^129,130^ to include diverse similarity algorithms like SIMILE,^131^ entropy similarity,^132^ neutral loss matching,^133^ reverse cosine,^134^ Spec2Vec,^135^ MS2DeepScore,^136^ and others,^137^ allowing detection of multiple ion modifications. Molecular networking is now integrated into tools like GNPS,^129^ MetaboScape, QIIME,^138^ Compound Discoverer, MatchMS,^139^ MZmine,^35^ NetID,^140^ MetDNA,^141^ KGMN,^142^ SGMN,^143^ MS-DIAL,^144^ MetGem,^145^ NP^3^,^146^ and implemented in R,^147–151^ Python,^152^ and even Rust.

Enhanced approaches like ion identity molecular networking (IIMN)^120^ and tools like NetID, MetDNA, KGMN, SGMN, and NP^3^ combine MS1 and MS/MS data—integrating retention time and peak shape driven approaches (like CAMERA or RAMClust) with spectral similarity. This allows clustering of ISFs with their precursors, reducing network redundancy and improving interpretation of complex data sets (Figure S1f). Additional strategies such as ISFrag^52^ and HERMES^187^ have leveraged peak correlation analyses and further improved ISF assignment accuracy by incorporating MS/MS spectral similarity—particularly with low-energy MS/MS data. The validity of this approach has been vindicated by a recently published study showing that in-source fragments such as water losses show fragmentation behavior very similar to their protonated counterparts.^153^

### There Are Large Differences in ISF Proportions Documented in the Literature

There is quite a bit of variation in the reported prevalence of ISFs across the literature, largely due to differences in experimental setups and the strategies used to calculate ISF percentages. ISF proportions have been reported in several ways: as a percentage of observed metabolites, as a percentage of detected peaks, or as the proportion of ions identified as ISFs from standard compounds (Table S1). When ISFs were quantified based on the number of detected peaks in biological extracts, reported values ranged from 2% to 35% with the majority below 10%. When calculated based on the number of metabolites, ISF proportions were in the same range with <11%. In contrast, studies using analytical standards reported substantially higher ISF rates, ranging from >1% to 70%.

Only one study—that we are aware of—has systematically examined ISFs by adduct type, showing that in the same data set, [M + H]^+^ ions yielded 67% ISFs, while [M + Na]^+^ ions produced less than 1%.^2^ More research is needed to determine whether these findings are generalizable across other adduct types and ion forms. However, a recent study demonstrating that alkali metal ion adducts generally led to less fragment ions even upon intentional CID fragmentation support these results.^153^ While the overall trend suggests—but does not 100% confirm this yet—that ISFs are more prevalent in standard mixtures than in biological samples even within the same study^52^—likely as a result of high standard concentrations and matrix free samples—it is also clear that ISF proportions cannot be generalized across metabolomics studies. Without standardized definitions and methodologies, comparisons across studies become inconsistent—effectively comparing apples to oranges.

Factors that influence ionization-related phenomena producing multiple ion forms beyond the intact protonated or deprotonated species, such as ISF, adduct- and multimer-formation include analyte concentration, molecular class, instrument type, salt content, source cleanliness, ambient humidity, analyst expertise (we were all beginners once), and—most importantly—instrument settings. Only some of these factors have been systematically studied. Instrument conditions are among the most influential contributors to ISF formation.^55^ To mitigate this, some mass spectrometry vendors have developed “soft” ionization methods that reduce ion activation energy during ion transfer. These approaches are used in fields such as native mass spectrometry, where preserving noncovalent interactions (e.g., iron–sulfur clusters, protein–ligand complexes, multimeric proteins, entirely intact viruses) is essential.^154–156^ Under these “detuned” conditions, low-energy transfer settings minimize fragmentation but can also reduce signal intensity, creating a potential trade-off between suppressing ISFs and maintaining sensitivity. However, studies have shown that this trade-off can be largely mitigated. For example, Criscuolo et al.^55^ demonstrated across multiple lipid classes and three Orbitrap instruments that ISF can be minimized with negligible effects on signal-to-noise ratio.

Analyte structure and its chemical properties, including metal chelation, strongly influence ion behavior during ionization. Even at 0 V nominal CID settings, residual ion transfer energy—typically a few volts—can cause limited fragmentation. While this energy is often insufficient to fragment more stable molecules, compounds containing labile bonds or functional groups such as phosphodiesters, phosphate, thioester, thiol, and glycosidic linkages require little energy to fragment and are therefore more susceptible to ISF formation. For instance, molecules like artesunate produced as many as 7 ISFs while no ISFs could be detected for corylifolinin under the same experimental conditions (Figure 5).^51^ Similarly, molecules that form stable adducts with metals such as Na^+^ typically require more energy to fragment, making them less susceptible to ISF. Functional groups can also modulate specific fragmentation pathways—for instance, hydroxyl groups often promote water loss during ionization.^153,157^

**Figure 5:**
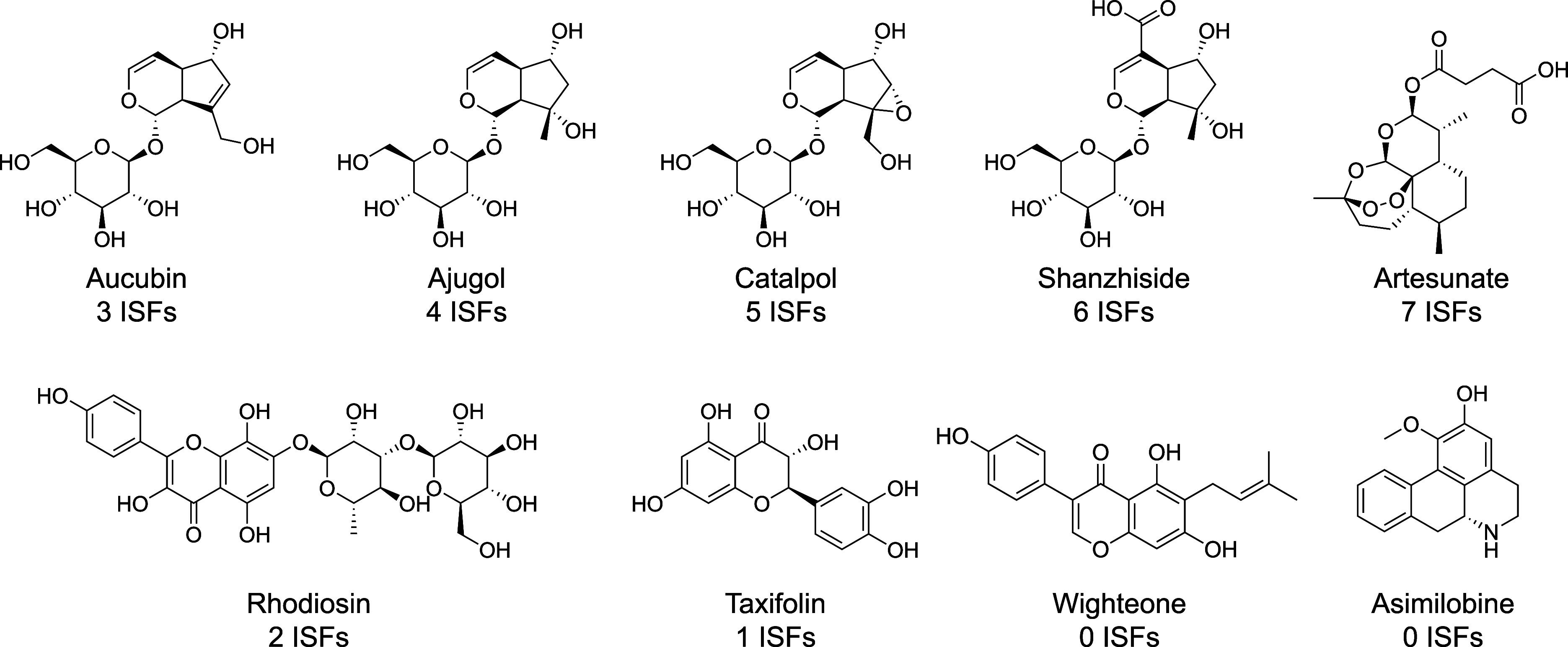
ISFs are molecule specific. Numbers of reported ISFs for various small molecules as reported in a standard mixture under one experimental condition by ref 51.

## How Big of a Problem Are ISFs in Metabolomics Studies?

A review of over 100 untargeted metabolomics papers published in 2024 indicates that fewer than 2% of biological studies explicitly mention in-source fragments (ISFs), whereas ISFs are referenced more frequently in methods-focused publications. This pattern suggests several possibilities: ISFs may be successfully minimized under common experimental conditions, effectively handled by existing software tools, or may not critically interfere with the ability to answer core biological questions. To date, there is no documented evidence of widespread or systematic misinterpretation due to ISFs that significantly alters biological conclusions. However, while writing this manuscript, a study was published describing misannotations of glycosylated molecules caused by unrecognized ISFs, highlighting the potential for such errors in specific compound classes.^53^

### Using ISFs in Annotations with Spectral Reference Libraries

We advocate for the inclusion of MS/MS spectra of ISFs in all spectral reference libraries. These spectra, which resemble MS^3^ or pseudo-MS^3^ data, can significantly aid in metabolite annotation. While major spectral libraries such as NIST,^158^ GNPS,^129^ MoNA,^159^ mzCloud,^160^ and MassBank^161^ all contain data from ISFs, and their inclusion is valuable. The largest proportion of ISF-containing spectra is found in NIST 2023, with approximately 58% of MS/MS records originating from ISF precursors. However, this likely captures only a subset of the full range of ISFs that molecules can undergo. Among those included, the most common ISFs included in reference libraries are neutral losses such as water and ammonia. Expanding spectral libraries to systematically include as many ISF-derived MS/MS spectra as possible would significantly enhance their utility. It would allow for the direct recognition of ISFs through spectral matching, ultimately improving annotation robustness and minimizing misidentification in complex data sets.

While there’s a risk of misassigning true molecular ions as ISFs in some cases (e.g., Figure S1c), the overall benefit is a broader and more flexible annotation framework that is valuable in untargeted metabolomics, where diverse ionization behaviors and analytical conditions are common. ISFs as part of MS/MS libraries can also offer annotation opportunities. As fragments of precursor molecules, they contribute complementary structural information that can increase confidence in metabolite assignments. For example, consider an MS/MS match from a reference library to ornithine ([M + H]^+^, *m*/*z* 133.0972). If an additional MS/MS match is observed for a coeluting signal at *m*/*z* 116.0706—an ISF corresponding to the loss of an amine (−NH_3_), effectively forming a proline-like structure ([M – NH_3_ + H]^+^)—this second match would provide orthogonal evidence supporting the annotation of ornithine (Figure S1d,e). However, if the MS/MS spectrum and retention time (or ion mobility drift time) of the *m*/*z* 116.0706 feature instead aligns with proline, also an endogenous metabolite, it would be reasonable to hypothesize that the signal originates from proline rather than from an ISF of ornithine. This hypothesis should be experimentally validated using authentic standards.

Consequently, MS/MS libraries should ideally contain both precursor and ISF fragmentation patterns for metabolites, enabling simultaneous testing of both possibilities. Although such coverage is currently limited, we see considerable untapped potential in systematically leveraging ISF-derived substructures—together with MS^*n*^ fragmentation—to drive metabolite discovery. Recent stepped-energy MS^3^ studies further support this concept, showing that ISF-derived fragments can serve as meaningful structural leads for identifying previously unrecognized metabolites.^162^

### Scenarios Where ISFs Are Informative

In addition to their value within reference libraries for MS/MS annotation, ISFs offer an underutilized opportunity for structural annotation in data sets lacking fragmentation spectra—such as MS1-only experiments or imaging mass spectrometry (MS) acquired without MS/MS. In these contexts, ISFs can enhance annotation confidence by providing substructure-level information directly from full-scan data. For example, after deconvolution of ion forms—adducts, isotopes, and multimers—fragments with recognizable substructures can be identified. Characteristic ISFs such as phosphate groups from phospholipids or glycosidic cleavages in glycoconjugates can immediately suggest chemical motifs and provide clues to molecular identity (Figure 6a). These fragments act as diagnostic signals that can inform chemical class assignment or suggest candidate structures, even in the absence of tandem MS.

**Figure 6:**
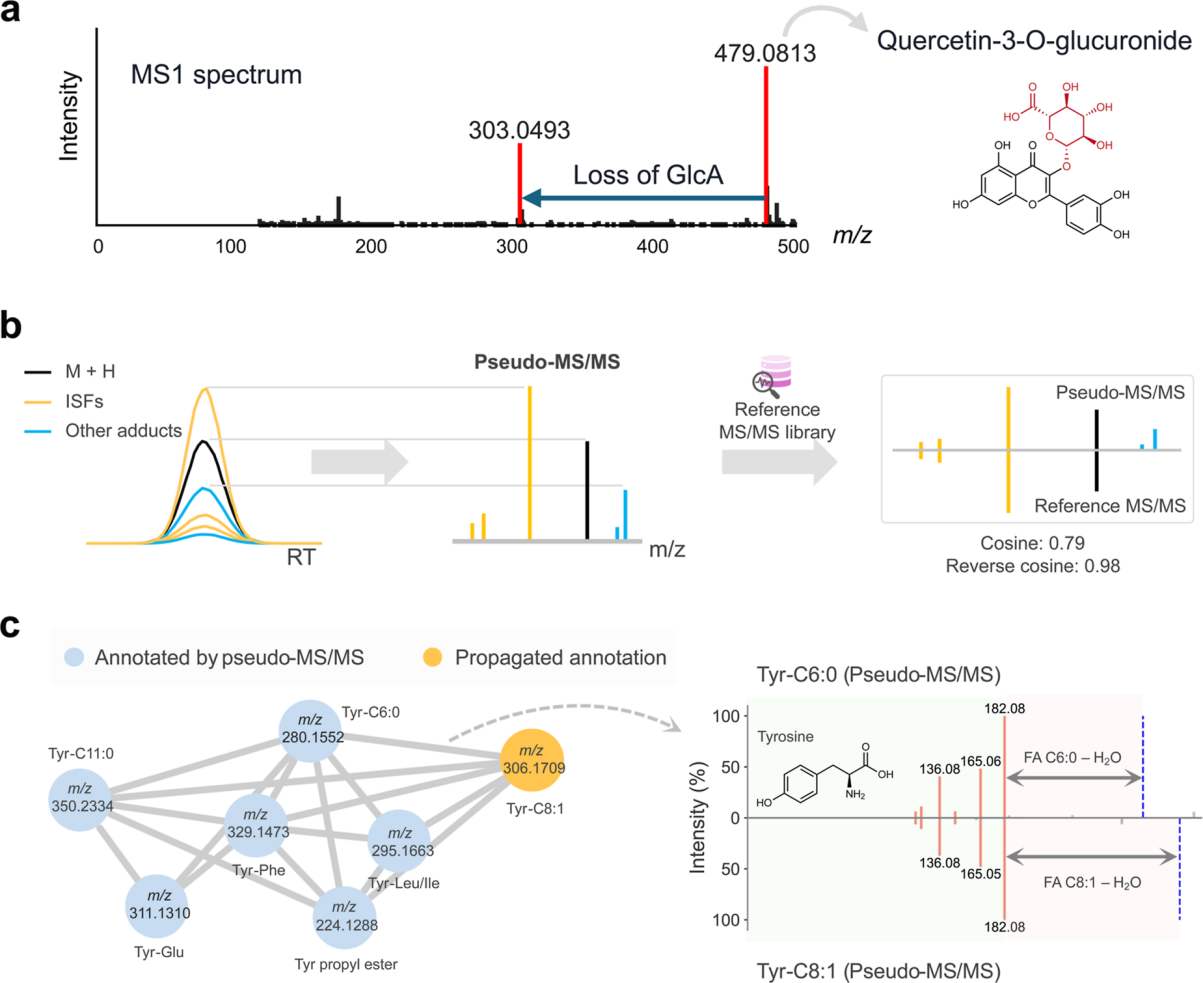
Leveraging beneficial aspects of ISFs—ISF-based (sub)­structure annotation and molecular networking. (a) ISFs can suggest chemical motifs. (b) Generation of pseudo-MS/MS spectra and structure annotation. (c) An example molecular network generated using pseudo-MS/MS spectra in the IBD data set. Tyrosine-related compounds were annotated and linked. The mirror plot shows pseudo-MS/MS spectra from Tyr-C6:0 and Tyr-C8:1. Adapted from ref 167.

This potential can be more systematically explored by deconvoluting ions that share chromatographic peak shapes and retention times to generate a pseudo-MS/MS spectrum.^125,163–166^ Here, peak height/area intensity serves as a proxy for fragment abundance. These pseudo-MS/MS spectra can be directly searched against spectral libraries, particularly for labile molecules, as demonstrated^125,163–168^ (Figure 6b). ISF was shown to be structural class specific. However, widespread annotation remains limited because most public reference library spectra are acquired at higher energies—typically in the range of 20–65 eV—which do not resemble the lower-energy ISFs produced in-source. One solution is to deliberately increase the in-source energy to a level where ISF-derived pseudospectra more closely resemble true MS/MS reference spectra, thereby improving spectral matching and annotation accuracy.^169^ Nevertheless, this approach comes with a caveat: since pseudo-MS/MS spectra are still derived from MS1 data, they often include fragments originating not only from [M + H]^+^ or [M – H]^−^ ions but also from other adducts or multimers. This compositional heterogeneity can dilute the quality of the match and reduce spectral similarity scores if not accounted for.

To address this, reverse cosine scoring—where only the ions present in the reference MS/MS spectrum are used for matching^134^—has proven effective in annotating pseudo-MS/MS spectra despite the noise introduced by unrelated ions.^167^ Still, ISF-based annotations inherently carry lower confidence than those derived from dedicated MS/MS experiments due to the lower-energy conditions under which ISFs form, which typically generate fewer diagnostic fragments, particularly in the low *m*/*z* range. One proposed approach to partially address this limitation involves adjusting the intensity distribution in spectral libraries—e.g., through peak intensity scaling—by down-weighting low *m*/*z* fragments and amplifying higher *m*/*z* ions to better align with the fragment distribution seen in ISF-derived pseudo-MS/MS spectra.^170^

Although these strategies cannot substitute for the confidence gained from direct MS/MS acquisition, they offer a unique and valuable path forward for reanalysis of MS1 data, especially considering that >40% of public metabolomics data in data repositories is MS1-only. As spectral libraries expand, these approaches create new opportunities to annotate legacy and imaging MS data sets beyond conventional MS1-based workflows, such as those described in the MS1-based FDR-controlled annotation framework by Alexandrov et al.^171^ Just like regular MS/MS, pseudo-MS/MS can also be subjected to molecular networking for visualization and annotation propagation (Figure 6c).

### Interferences of ISFs in Statistical and Machine Learning-Based Prioritization

Despite the widespread use of statistical and machine learning techniques such as PLS-DA,^172^ random forests,^173^ and LASSO^174^ in metabolomics, we found limited information on how ISFs and other ions may impact statistical outcomes when applied to ion peak abundances. We also consulted peers in the field and were surprised by the lack of focused studies that could be identified.^175,176^ This gap suggests one of two possibilities: either ISFs are not perceived as a major problem, or their influence is underappreciated because we lack strategies to systematically and routinely leverage them. The reality is likely a mixture of both.

While ISFs are occasionally mentioned in passing, we found no comprehensive studies examining their direct effects on statistical outcomes and only one study addressing this at all (see discussion in the next paragraphs). However, some impacts can be postulated from the characteristics of applied statistical methodologies. This is due to the fact that ISFs are not independent biological variables, but derivatives of parent ions, generated during ionization. If these fragments are treated as independent variables—i.e. not grouped by methods discussed above—the impact will depend on the applied statistical methodology. In univariate analysis (e.g., *t*-test or ANOVA) multiple testing correction via Bonferroni or Benjamini–Hochberg^177^ will—assuming that all observed metabolites have an equal chance of being affected by ISF—lead to decreased statistical power as it penalizes higher numbers of tested variables. When interpreting statistically significant features (e.g., based on the volcano plot in Figure 7) care must be taken not to conflate the number of significant features with the number of significant metabolites, as different ion species of the same molecules tend to show similar statistical trends, though not always consistently. ISF abundances often correlate partially with their precursor ions, though not in a predictable or linear fashion. This may lead to (i) redundant selection of both parent and ISF, suggesting multiple distinct discriminant features; (ii) erratic ISF behavior, as reported by ref 178, where ISFs do not scale linearly with concentration (as opposed to protonated and potassium adducts), which may cause spurious group separation or distorted variable importance rankings.

**Figure 7:**
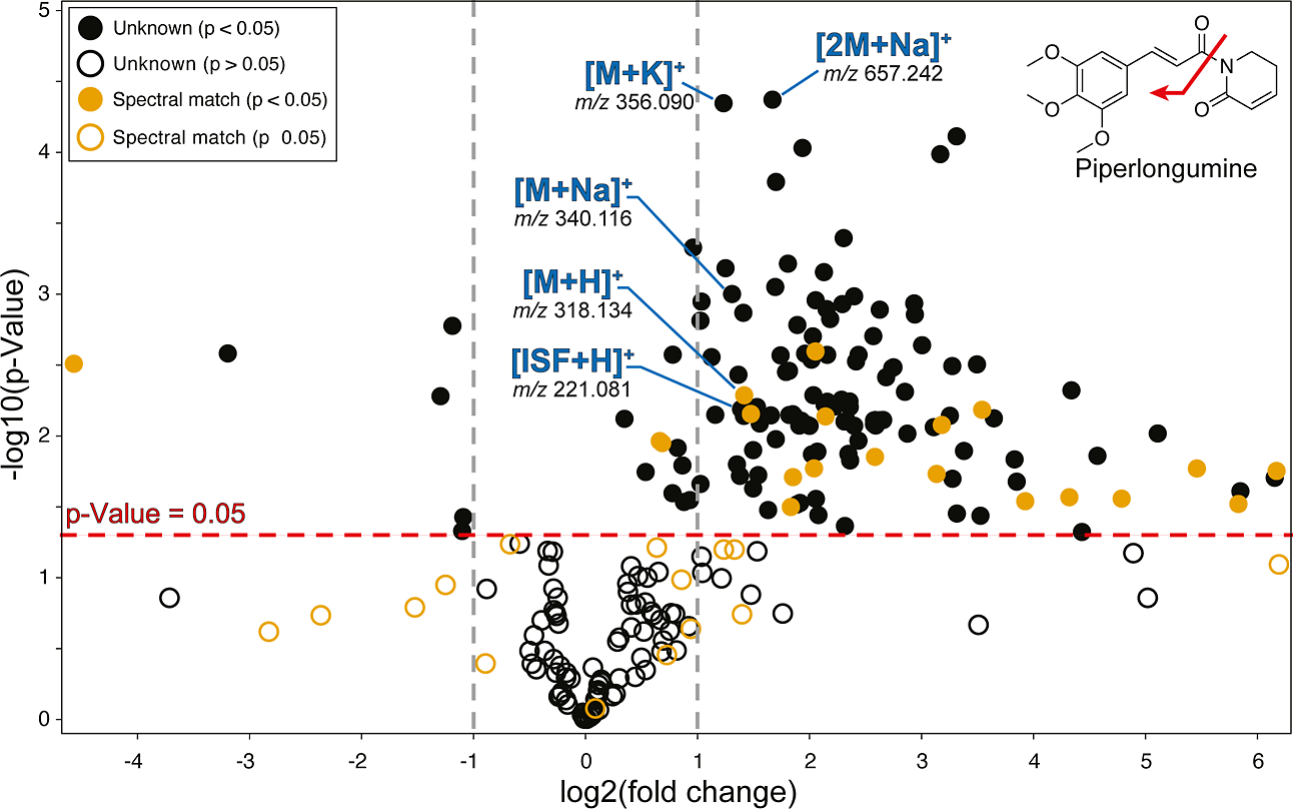
Multiple ions and ISF originating from the molecule often follow the same direction with respect to fold change but can vary statistically. Piperlongumine structure is shown in the upper right corner of the plot (red arrow represents where the molecule fragment *in source*). Horizontal dashed red lines in the plot represent a *p*-value of 0.05. Vertical dashed gray line represents down- or up-regulation by a factor of 2. The red arrow indicates where the molecules fragments to generate the ISF.

Although ignoring them does not hinder many metabolomics applications, accounting for all ion forms of the same molecule may influence most statistical analyses. While this effect has not been systematically examined for most statistical approaches, one study using ANOVA-type Bayesian modeling of covariate effects reported that including multiple ion species of the same compound improved statistical power.^179^ Although based on a small sample size, the study suggests that ISFs and other ion forms may not only be underappreciated as sources of redundancy, but also underexploited as potentially informative signals. In practical terms, failing to group or merge ion forms may cause valid biological signals to fall just short of statistical significance—thus potentially missing biomarkers that fail to cross the conventional *p* = 0.05 threshold. If this finding generalizes to other statistical frameworks, and particularly with larger sample sizes, then improvements in statistical sensitivity might be achieved not only by increasing sample numbers, but also by incorporating knowledge of ion chemistry to systematically group features from the same metabolite.

In principle—but not thoroughly assessed in the literature—multivariate unsupervised techniques such as principal component analysis (PCA) or clustering based on Euclidean distances between features the impact will depend on the selected methodology. PCA will inherently collapse correlated variables into the same principal component keeping the impact of duplicated variables will be minimal. In feature distance based clustering techniques (often assessed through heatmaps with dendrograms) more care should be taken by applying distance metrics accounting for correlation (e.g., Mahalanobis distance^180^) or grouping ion species upfront. However, we want to emphasize that these effects have, to our knowledge, not been studied in detail for untargeted metabolomics data. The same is true for multivariate supervised methods like PLS-DA,^172^ random forest,^181^ or LASSO.

As has been shown, salt content, matrix composition and concentration effects can alter the formation of ISFs.^120,178^ Therefore, the same biological sample processed under different ionization conditions due to the presence of the salts may yield different ISF patterns, introducing effects that are not due to underlying biology. Some of these differences in salt concentrations in biological samples could potentially even arise from biological differences within a single cohort. One could envision that a person with cystic fibrosis that has a mutated potassium transporter will have a significantly altered salt profile in their samples when compared to samples from healthy individuals. So far it is unknown if such biological variables indeed alter ionization properties and susceptibility to ISF formation. In addition, normalization techniques (e.g., total ion count, probabilistic quotient normalization) assume that features are independent and identically distributed. ISFs violate this assumption by being structurally and behaviorally dependent on other features. This can, in principle, skew normalization baselines and reduce model generalizability. However, the on average relatively low intensity of ISFs compared to their precursors^2^ likely reduce this effect of this and the real extent remains to be assessed. Different from targeted metabolomics studies where stable isotope labeled standards can be utilized to correct for such effects, untargeted metabolomics requires different approaches.

To solve these issues, grouping ion features derived from the same molecule prior to statistical analysis has been proposed as a strategy to reduce redundancy and improve interpretability. Tools such as MS-FLO,^119^ CAMERA,^115^ and CliqueMS^117^ and other referenced above were specifically designed to group related ion forms—primarily adducts—and to remove isotopes to avoid double counting. While some of these tools do not currently handle unexpected ISFs, we believe they could be readily extended or adapted to include ISF detection and grouping. However, while grouping related ion features holds promise for enhancing statistical robustness and reducing artifactual signals, it also introduces challenges. Differences in ionization efficiency and detection sensitivity across ion forms (e.g., between [M + H]+, adducts, and ISFs) can complicate feature alignment and quantitative interpretation.

In summary, the influence of ISFs on statistical analysis in metabolomics may be undervalued, not because their impact is negligible, but likely because systematic tools to detect and account for them are underused and generally not part of the packages and software used for statistical analysis. Incorporating ISF-aware preprocessing—such as ion deconvolution or network-based grouping—may even improve results beyond what would be possible without the consideration of multiple ion species.

## How Do ISFs Impact Discovery of New Molecules?

To date, we are not aware of any published new molecular structure that was incorrectly proposed as a result of an ISF, and if such cases exist, they are exceedingly rare. Fundamentally, ISFs will not typically lead to the erroneous reporting of novel structures because structural elucidation workflows rely on a wide array of orthogonal validation methods that extend well beyond mass spectrometry. These methods include compound isolation followed by orthogonal methods such as 1D and 2D nuclear magnetic resonance (NMR), X-ray crystallography, electron diffraction (ED), circular dichroism (CD), atomic force microscopy (AFM), infrared (IR), and ultraviolet–visible (UV–vis) spectroscopy.^182^ Liquid chromatography is routinely used to confirm retention time alignment with authentic standards, after the compounds have been synthesized, including chromatography comigration experiments, MS/MS matching and/or ion mobility spectrometry (for drift time matching).

In addition, specialized techniques such as the crystalline sponge method—which uses porous metal–organic frameworks (MOFs) to enable X-ray structure determination without crystallizing the target compound—further expand structural elucidation capabilities. Degradation-based strategies, such as Marfey’s analysis for determining amino acid stereochemistry, along with other chemical derivatization or hydrolysis techniques, help dissect substructures and establish absolute stereochemical configurations. Stable isotope labeling, genetic perturbation experiments (e.g., knockouts or overexpression), biosynthetic gene cluster analysis, in silico prediction tools, and total or partial chemical synthesis all provide further layers of confirmation. By the time a new structure is confidently proposed, it is typically supported by multiple, independent lines of evidence. Even in the rare cases where a structure is later revised following total synthesis, such corrections are not linked to ISF interference. Altogether, while it is theoretically possible for ISFs to complicate structural analysis, the many orthogonal layers of modern structure elucidation pipelines makes mischaracterization due to ISFs highly unlikely.

### Why Annotating as Many Ion Features as Possible Helps, Irrespective What They Are

It is incorrect to assume that all unannotated ion features are new molecules. On the flipside of the coin we also cannot assume that most ion features are artifactual and/or useless junk. We would argue that the goal should be to annotate all ions in an LC–MS/MS experiment, even if this goal is still far away.^17,87,183^ Once annotated, then one can make an informed decision on how one wants to handle that mass spectrometry feature.

Aside from only annotating the [M + H]^+^ (or [M – H]^−^) ions as being relevant, many other ion forms can provide value. Metal adducts, for example, can provide valuable biological insight, such as revealing metal-binding properties that are essential to molecular function.^155^ ATP requires magnesium, heme requires iron, and such adducts are observed in LC–MS/MS data.^184,185^ Although mass spectrometrists often find Na^+^ or K^+^ adducts an unwanted nuisance, it is known that some fatty acids require complexation with Na^+^ for transport.^186^ Noncovalent multimeric species, too, are conceivably an overlooked component of the functional metabolome; just as proteins function in multimeric complexes, perhaps some small molecules do as well—we simply do not know yet. Unfortunately there is not yet a systematic framework that exists to tell if an adduct or a multimer is biologically relevant or not—and represents an important research opportunity. Similarly, mass spectrometry phenomena, such as ISFs can give clues for needing to adjust the experimental setup to reduce them. However, ISFs can also offer structural clues, and give insight into molecule reactivity and stability and there are experiments where ISFs are desired.^169,170^

Beyond ISFs, other seemingly unwanted features—such as background signals from plastics or solvents, or characteristic patterns like sodium formate clusters (commonly observed when formic acid is used as a mobile phase additive)—also hold value. Proper identification of these signals through robust quality control (QC) practices is essential. Annotating such ions can inform adjustments to sample handling, chromatographic conditions, or ionization settings, and in some cases, may even inspire the development of new experimental workflows better suited for extracting biologically relevant information.

An example of such an experimental workflow that leverages knowledge about ion forms as part of the experiment is to bias instrumental data acquisition is HERMES. HERMES, a molecular formula-oriented method that optimizes MS/MS acquisition by prioritizing ions most likely to be biologically relevant.^187^ Based on structural databases HERMES systematically selects plausible ions in the raw LC–MS data for MS/MS acquisition—while excluding background contaminants, isotopes, redundant adducts, and ISFs. This targeted prefiltering enhances the biological specificity of MS/MS acquisition, resulting in improved annotation rates of biologically relevant ions. Notably, the number of confident metabolite identifications achieved with HERMES was 3 times higher than those obtained using commonly used iterative data-dependent acquisition (DDA) workflows as it eliminated MS/MS acquisition time on ions that are not sample-specific—such as background signals or redundant ion forms.

In short, even the so-called “junk” in mass spectrometry carries meaningful information and can be leveraged. The real challenge—and opportunity—is to develop systematic frameworks that allow us to harness this complexity, turning noise into knowledge by extracting value from all detectable ion forms.

### The Role of Public Data Sharing in Improving Transparency, Data Knowledge Reuse and Reproducible Science, Including for Assessment of ISFs and Other Ion Forms

Over the past decade, publicly accessible metabolomics data has grown exponentially and there are multiple metabolomics or generalist repositories available to the community.^129,188–192^ This surge reflects not only funding mandates and evolving norms around transparency and reproducibility, but also a broader realization—one that echoes key lessons from the sequencing field in the late 1980s and early 1990s. Back then, the high cost of sequencing technologies sparked intense debate over how to maximize scientific investment return. A central argument was that publicly funded data should not be siloed or discarded, but instead deposited in accessible repositories to avoid redundancy and enable reuse for future, unforeseen research questions.^193^ These discussions led to the establishment of GenBank and the adoption of open-data principles codified in the Bermuda Principles and Fort Lauderdale Agreement,^194,195^ ultimately transforming sequencing into a scalable, collaborative, and data-centric scientific enterprise.

Metabolomics is now approaching a similar inflection point. Despite billions of dollars in public and foundation investment,^196^ only a small fraction of generated metabolomics data is typically analyzed, with the rest often going unused. This underutilization represents not only a technical gap but a missed scientific and economic opportunity. There is growing recognition that archived metabolomics data—particularly when harmonized and made machine-actionable^86,139^ —can drive new discoveries across clinical, environmental, and biological domains, including for questions that were never envisioned in the original studies. As a result, large-scale reanalyses involving billions of spectra are emerging as a defining and transformative direction in untargeted metabolomics.^66,93,197–199^

Public data sharing plays a foundational role in improving transparency, enabling data reuse, and advancing reproducible science—including when it comes to assessing ISFs and other ion forms such as adducts and multimers in untargeted metabolomics. ISF formation is influenced by many factors, including ionization conditions, instrument design, sample composition, and analyte class, making isolated experiments insufficient for drawing generalizable conclusions. By making raw and processed LC–MS/MS data sets publicly available, researchers can begin to systematically compare across diverse experimental conditions, instruments, and sample types to identify consistent patterns, outliers, and context-dependent behaviors of ISFs. This collective knowledge—despite having room for enormous improvement—accelerates the development of the best computational methods to flag, annotate, and deconvolute ISFs and related ion forms—ultimately improving annotation accuracy and minimizing false discoveries. Public data also enables independent validation, and supports the discovery of previously unrecognized ionization behaviors. Importantly, it empowers the broader community to challenge or confirm published findings, contributing to open, self-correcting science.

In the context of ISFs—where interpretation can vary and implications extend to debates about the scale of the dark metabolome—transparent access to data ensures that conclusions are grounded in reproducible, community-accessible evidence rather than single-laboratory nonverifiable observations.

### Repository-Scale Analysis: ISFs in a New Era of Discovery Using over a Billion MS/MS Spectra

Although public data sets still represent less than 1% of all metabolomics studies, efforts to harness them at scale are accelerating with many studies conducting repository level analysis (Supporting Information 1).^200^ As of October 2024, the GNPS/MassIVE ecosystem—along with repositories like MetaboLights and Metabolomics Workbench—hosts approximately 6000 public studies, nearly 2 million LC–MS­(/MS) files, and over 2 billion MS/MS spectra, a figure that is doubling every 2–4 years. These data sets are now being used to construct biochemical atlases of human fluids and tissues,^93,201–203^ and to power search engines that link metabolites to their sources in foods, tissues, drugs, and the microbiome.^204–207^ Mining data at this scale has only recently become technically feasible, and it holds great promise to transform how metabolomics research is conducted and interpreted. However, this potential can only be fully realized if care is taken to properly recognize and handle analytical phenomena such as in-source fragments, adducts, and background contaminants.

Tools such as MassQL,^208^ MASST,^209,210^ and panReDU^86^ support structured queries and reverse metabolomics,^197,211^ enabling searches across public data sets. However, continued growth in data scale and complexity demands better infrastructure: faster searches, improved clustering, scalable indexing, provenance tracking via USIs,^86,209,212–214^ and increasingly, foundation models.^199^ As repository-scale metabolomics becomes more feasible, understanding ion forms—particularly ISFs—and knowing when they matter becomes increasingly important. In some contexts, ignoring ISFs is acceptable; in others, it may lead to incorrect conclusions or missed discoveries.

To illustrate how large-scale reanalysis of public data sets can drive metabolite discovery while accounting for ion forms, we present four case studies. These examples show when it is critical to annotate ISFs, especially in studies that process billions of spectra. Ultimately, community guidelines will be essential to standardize how ISFs and other ion forms are handled at scale. Until then, predictions from repository-scale analyses must be experimentally validated when identifying novel molecules. Representative case studies or repository scale analysis and how they dealt with ISFs and other ion forms are provided in Supporting Information 1.

### Uncovering the True Scale of the Dark Metabolome Requires the Analysis of Many Samples, Sample Types and Conditions

Although a single sample may already contain a substantial number of unannotated metabolites, the global dark metabolome is vastly larger. Most blood, plasma, or serum samples analyzed in metabolomics studies are collected under fasting conditions. While useful for reducing variability, these conditions poorly reflect the full spectrum of biological diversity—spatially, temporally, and in terms of environmental exposures. Human biology is shaped by highly dynamic processes, including rapid fluctuations in metabolite levels—some occurring in microseconds, such as shifts in lactate production during hypoxia—or following diurnal and circadian rhythms that can lead to changes of several orders of magnitude.^215,216^

Exposure signatures also vary greatly by matrix and time scale. For instance, after consuming a caffeinated beverage, caffeine and similarly other exposures can be detected in blood and even on the forehead within 5–60 min.^217^ However, it may take 1–12 h to appear in urine,^218,219^ 24 h to 2 weeks in feces, and days to weeks in hair or nails—where it can remain detectable for months or even years depending on how frequently they are cut.^220–224^ Beyond time, the type and abundance of molecules vary by life stage—from birth through aging and even post-mortem—and differ significantly across organs. While some molecular signatures are shared, a sample from the brain will contain a different metabolite profile than samples from the intestine, muscles, pancreas, thymus, lymph nodes, or bone marrow.^207,225^ Each organ exhibits unique temporal dynamics, ranging from subseconds to a lifetime.

Moreover, the vast majority of biological conditions have yet to be sampled using metabolomics, including with varied extraction protocols and ionization modes. Added to this complexity is the global diversity in human genetics and microbiomes, which further shapes the metabolome in ways we are only beginning to understand. In fact, the number of new structural scaffolds is ever expanding to this day.^226^ Capturing a much broader multidimensional biochemical diversity than that we currently understand is essential to fully illuminate the dark metabolome.

### Opportunities

While several research opportunities are highlighted in the perspective, there remain broad opportunities to better leverage and understand ion forms and ISFs for the benefit of the larger research community. The first is for software developers. Most metabolomics software still treat ISFs and other complex ion forms as nuisances to be removed prior to the discovery phase. However, as demonstrated throughout this manuscript, these so-called “redundant” ion forms can, in fact, provide valuable information and improve analytical outcomes.^155,166,179^ These examples are only scratching the surface of what is possible and we hope that this work will inspire further developments into this direction.

Furthermore, metabolomics laboratories typically operate in isolation, each accumulating unique knowledge about annotations—including ion forms and ISFs. Systematically capturing and depositing this knowledge alongside raw data in public repositories could revolutionize data reusability and the identification of ISFs. For this to be effective, annotation tables must be submitted together with the raw data and include precise links to the underlying spectral evidence informing those annotations. While standards like mzTab-M approach this goal, they lack a robust connection to raw data, which could be resolved by implementing identifiers such as MRI/USI links.^212,213,227^ Beyond improving reproducibility and transparency—cornerstones of rigorous scientific inquiry—such raw data provenance would accelerate discovery, facilitate cross-study comparisons, and enhance confidence in metabolite annotations. We anticipate that such a transition would fundamentally shift metabolomics data repurposing and transform how the entire field conducts annotation and analysis.

### Guidelines on Metabolite Discovery and the Importance of Careful Review

Peer review is essential for scientific progress. When this process falters, it can have significant consequences—not only for individual careers but for the advancement of the entire field. Sending the message that the metabolome is essentially “already discovered”, mostly “junk” or “artifactual”, undermines the field’s relevance and urgency, which is far from accurate. These false perceptions particularly affect the careers of our younger co-workers. We, therefore, believe it is essential to include dedicated guidance on how to responsibly review and evaluate discovery-based metabolomics studies. Especially for reviewers less familiar with the nuances of mass spectrometry data structure, it is easy to miss the broader significance of new findings amid concerns about artifacts. We propose a brief set of practical do’s and don’ts to support fair, informed, and constructive peer evaluation (Table 1).

**Table 1:** Do’s and Don’ts in Review of Papers and Grants Focusing on the Discovery of New Metabolites

DO	DON’T
**acknowledge the complexity of MS and MS/MS data.** Recognize that mass spectrometry data may contain ISFs, adducts, and multimer artifacts—but also that computational tools and experimental methods exist to distinguish these from genuine metabolites	**do not dismiss findings solely based on fragmentation concerns.** Avoid assuming that an MS/MS feature is an artifact without considering the full analytical and biological context, particularly when synthetic validation of the compounds or other lines of evidence are provided
**use first principles when applicable.** Not all metabolites ionize and fragment equally, *m*/*z* and MS/MS are proxies for structure, annotation is not identification, detectable features and molecules are not the same	**do not generalize from one data set or instrument.** ISF and ion behavior can vary across instruments, acquisition parameters, and compound classes. Avoid overgeneralizing conclusions about ions without considering these factors or broadly analyzing thousands of studies that are available in repositories
**support discovery-driven work.** Recognize that untargeted metabolomics can fuel two types of discoveries. It can implicate known metabolites in unexpected ways or it can be used to explore uncharacterized chemical space. Both aims have value and are foundational to metabolomics	**do not penalize studies for working in the dark metabolome.** The lack of a spectral match or database annotation does not invalidate a compound’s biological relevance. While it is fair to critique biological relevance, be open to novel chemical space, especially when the evidence is reproducible and interpretable. It is not uncommon that a molecule is discovered and the biological implication to be established years or even decades later
**request clarification, not rejection.** If uncertainty remains, request additional data or clarification rather than concluding prematurely that the finding is an artifact without proper evidence. Encourage transparency about limitations and confidence in the annotations and if dare can only be one molecule or represent multiple	**do not equate uncertainty with artifact.** Unidentified or partially characterized features should be viewed as opportunities for further study, not dismissed as noise unless clearly proven otherwise. However, the level of annotation confidence (MSI levels^228,229^ ) should be stated. Reviewers can and should ask for this information
**reflect on field-wide consequences.** Understand that dismissing emerging findings without due consideration may disproportionately affect early career scientists and slow down collective progress in metabolomics. Give them room to grow, guide them in the reviews—we were all early investigators once	**do not ignore physical constraints.** If a product ion is larger in *m*/*z* than its precursor of the same charge, it is not an in-source fragment. Misclassifying such ions undermines rigorous data interpretation. Such features should be further investigated, not dismissed as artifacts
**evaluate the totality of evidence of newly discovered molecules.** Consider orthogonal validation strategies such as MS/MS matching with synthetic standards, coelution, isotopic labeling, NMR characterization, and genomic context (e.g., biosynthetic gene clusters or known metabolic pathways)	**do not undervalue inter—data support.** When genomic, biochemical, or ecological context supports the presence of a novel metabolite, treat that as valid and complementary evidence—not as secondary to MS/MS matching alone. If you are not an expert in other supporting data types—make the editor aware to ensure they know how to find such reviewers
**promote reproducibility and provenance tracing.** If not available, ask for the code and underlying data to be made public. There are cases—such as human protections or intellectual property—where data/code is only accessible through restricted access. In such cases ask to describe how it can be made accessible. Making data and code transparent ensures that provenance of the annotations will be understood in the future	

## Conclusion

ISFs present both a challenge and an opportunity in metabolomics. When not properly considered, ISFs have the potential to confound molecular annotation and quantification but it has not prevented the many discoveries that metabolomics studies are providing. They also encode valuable structural information that can aid compound identification, clarify fragmentation pathways, and reveal related analogs or shared substructures. Rather than viewing ISFs solely as analytical artifacts, they can also be leveraged to illuminate aspects of the dark metabolome—the array of molecules detected in biological samples that are not yet annotated or understood.

This raises a broader scientific and philosophical question: how large is the dark metabolome? Or more expansively, how large is the metabolome of earth—or of the human population? The short answer is that no one knows. Still, researchers are beginning to offer conceptual frameworks and estimates. The total plausible chemical space of small molecules, whether synthetic or biologically derived, is estimated to range from 10^40^ to 10^60^ structures, of which less than 1% has been experimentally sampled.^230,231^

Emerging computational tools, including large language models, are starting to define which molecules within this space are biologically plausible with numbers in the billions.^25,79,232–235^ Some estimates suggest *a single individual* may be exposed to 1–3 million distinct molecules over a lifetime^236^—an idea captured in the concept of the “million metabolome,”^237^ which parallels the number of microbial genes found in a single person. Given the diversity in human exposure at the world population level—shaped by diet, lifestyle, environment, microbiome, and geography—the global human metabolome could plausibly reach tens of millions distinct molecules, possibly more. These numbers parallel the number of microbiome genes in the human population,^238–240^ not accounting for further expansion through metabolism. Yet, current databases capture only a fraction of this molecular diversity: approximately 20,000 molecules are part of metabolic maps like KEGG, 50,000 molecules have been reported in human blood through literature mining,^241^ around 30,000 detected metabolites are cataloged in the Human Metabolome Database,^242^ and another ∼220,000 small molecules are predicted or referenced elsewhere but not yet detected according to HMDB.^242^ This discrepancy underscores how much remains to be discovered.

Just as early explorers successfully navigated the world using hand-drawn maps, these tools worked—but they lacked precision. The advent of satellite-based navigation revolutionized our ability to traverse the globe with accuracy and confidence. Similarly, the metabolic maps we use today—largely hand drawn constructs created between the 1940s and 1970s—have provided a valuable foundation, but they remain inherently limited in scope and resolution. To truly navigate the complexities of human metabolism and the dark metabolome, we must transition to more accurate, data-rich, and adaptive representations—akin to moving from hand-drawn maps to satellite-enabled navigation. Fortunately, recent advances in data access,^213,243^ integrative software,^122^ foundation models,^199^ large-scale molecular networking,^244^ repository-scale analyses^86^ and search engines,^208–210^ and generative AI^25,79^ are beginning to provide the tools needed to bridge this gap. Together, these technologies ***and in combination with new and creative ways of thinking*** about metabolomics offer promising strategies to map, interpret, and ultimately understand the full breadth of the metabolome—including the darker corners we have only just begun to explore.

## Supplementary Material



## Notes

Figure S1: strategies to group ISFs and other ion types. Figure S2: leveraging ISF in repository scale analyses. Table S1: reported ISF percentages as derived for different methods and sample types. Supporting Information 1: case study 1: bile acids; case study 2: *N*-acyl lipids; case study 3: drug analog library. References (PDF)
