# Supplementary material for: A Perspective on Unintentional Fragments and Their Impact on the Dark Metabolome, Untargeted Profiling, Molecular Networking, Public Data, and Repository Scale Analysis
Source: JACS Au. 2025 Dec 1;5(12):5828–50. doi: 10.1021/jacsau.5c01063 (PMC12728610; doi:10.1021/jacsau.5c01063)
Supplement: Supplementary file 1 [file au5c01063_si_001.pdf]

Supporting Information for Publication.

Authors: Yasin El Abiead[1], Ipsita Mohanty[1], Shipei Xing[1], Adriano Rutz[2], Vincent Charron-Lamoureux[1], Tito Damiani[3], Wenyun Lu[4], Gary J. Patti[5], Nicola Zamboni[2], Oscar Yanes[6,7], Pieter C. Dorrestein[1,8,9,*]

*Corresponding author

Addresses:

1. Skaggs School of Pharmacyin a and Pharmaceutical Sciences, University of California San Diego, 9500 Gilman Drive, CA 92093-0751 San Diego, United States
2. Institute for Molecular Systems Biology, ETH Zurich, Otto-Stern-Weg 3, 8093 Zurich, Switzerland
3. Department of Biochemistry of plant specialized metabolites, Institute of Organic Chemistry and Biochemistry of the Czech Academy of Sciences, Flemingovo náměstí 542/2, 160 00 Prague, Czech Republic
4. Lewis Sigler Institute for Integrative Genomics and Department of Chemistry, Princeton University, Princeton, NJ 08544, United States
5. Department of Chemistry, Genetics, and Medicine, and Center for Mass Spectrometry and Metabolic Tracing, Washington University, St. Louis, MO 63110, United States
6. Department of Electronic Engineering & IISPV, Universitat Rovira i Virgili, 43007 Tarragona, Spain
7. CIBER de Diabetes y Enfermedades Metabólicas Asociadas (CIBERDEM), Instituto de Salud Carlos III, 28029 Madrid, Spain
8. Collaborative Mass Spectrometry Innovation Center, Skaggs School of Pharmacy and Pharmaceutical Sciences, University of California San Diego, La Jolla, CA, United States
9. Center for Microbiome Innovation, University of California San Diego, La Jolla, CA, 92093, United States

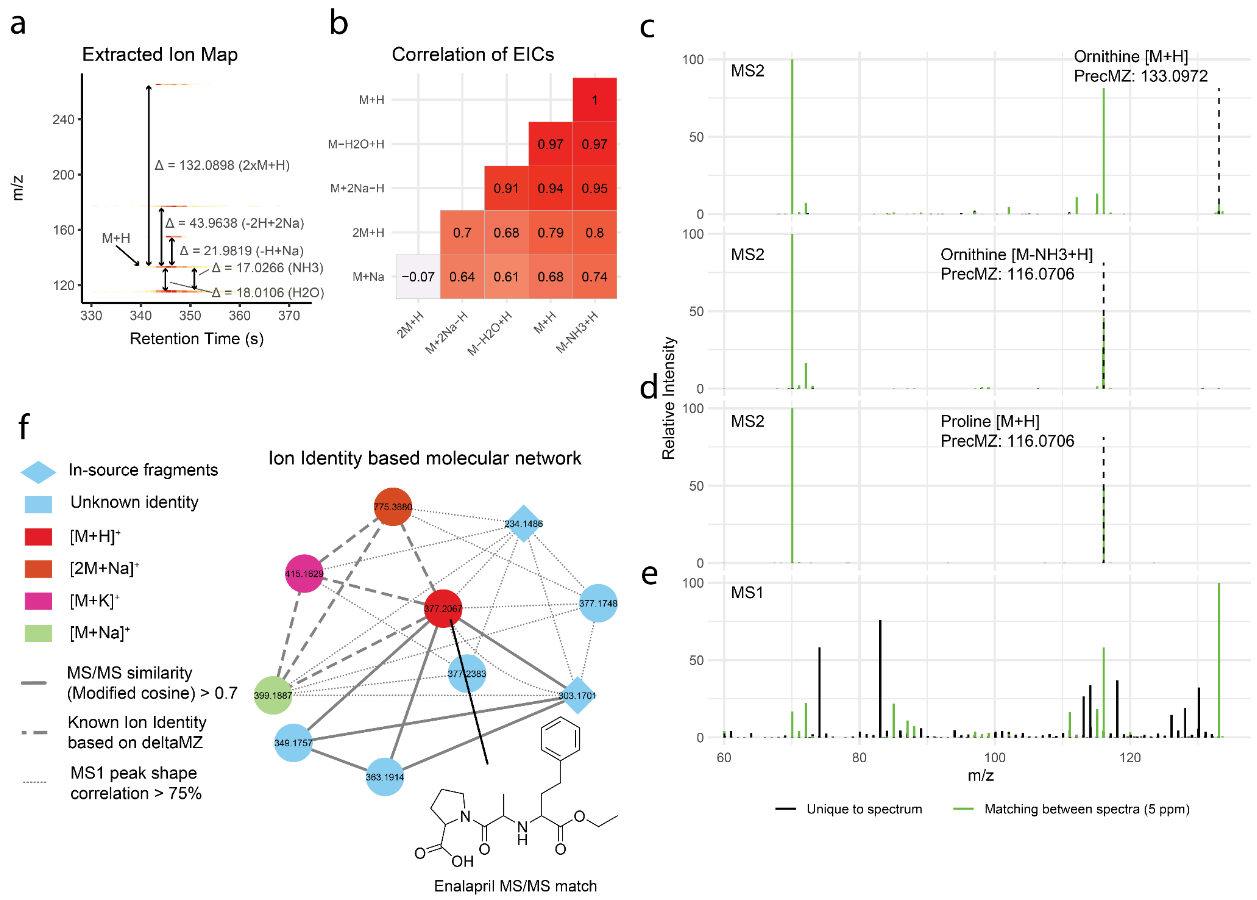

Supplementary Figure 1 | Strategies to group ISFs and other ion types. a) Utilizing co-elution and mz-distances corresponding to commonly observed ion forms, many ISFs and adducts can be grouped. b) At low intensities the peak shape correlation can fail, as can be seen here for the 2M+H and M+Na adducts, which serves as a powerful example of how peak shape correlation analysis can fail at grouping related features at low signal/noise ratios. However, it is possible to apply this strategy hypothesis-free – not requiring commonly observed *m/z*-differences. c) Utilizing MS/MS similarity between the MS/MS of the precursor ion and suspected ISF the relationship between ions can be confirmed. c) It should be noted that ISFs can produce MS/MS spectra very similar or identical to MS/MS spectra of metabolites. e) Utilizing the overlap between the MS/MS of precursor ions and the MS1 scan potential ISFs can be postulated. However, these should be confirmed through peak shape correlation (link). (f) Ion Identity Molecular Networking adds edges based on peak shape correlation and known mz-distances. Thereby common adducts can be identified, and other ion species potentially originating to the same molecule can be grouped.

Supplementary Table 1. Reported ISF percentages as derived for different methods and sample types.

Sample type	LC	MS	polarity	ISF [%]	Reference population	Reference
S. cerevisiae	HILIC	Orbitrap (Q-Exactive	pos	2%	Number of biological peaks that get labeled	(L. Wang et al. 2019)

		Plus)				
S. cerevisiae	HILIC	Orbitrap (Q-Exactive Plus)	neg	3%	Number of biological peaks that get labeled	(L. Wang et al. 2019)
E. coli	HILIC	Orbitrap (Q-Exactive Plus)	pos	6%	Number of biological peaks that get labeled	(L. Wang et al. 2019)
E. coli	HILIC	Orbitrap (Q-Exactive Plus)	neg	6%	Number of biological peaks that get labeled	(L. Wang et al. 2019)
E. coli	RPC	Orbitrap (Q Exactive Plus)	pos	8%	Number of detected biological peaks after isotope and charge carrier grouping	(Mahieu and Patti 2017)
mouse liver	HILIC	Orbitrap (Q-Exactive Plus)	pos	11%	Number of detected peaks	(Lu et al. 2020)
mouse liver	HILIC	Orbitrap (Q-Exactive Plus)	neg	9%	Number of detected peaks	(Lu et al. 2020)
NIST human plasma	RPC	qToF (Bruker Impact II)	pos	5%	Number of detected peaks	(Guo et al. 2021)
NIST human plasma	RPC	qToF (Bruker Impact II)	neg	2%	Number of detected peaks	(Guo et al. 2021)
NIST human plasma	HILIC	qToF (Bruker Impact II)	pos	7%	Number of detected peaks	(Guo et al. 2021)
NIST human plasma	HILIC	qToF (Bruker Impact II)	neg	4%	Number of detected peaks	(Guo et al. 2021)
Meta-analysis of 61 public dataset	multiple	multiple	both	<10%	Number of detected peaks	(Chi, Mitchell, Zheng, et al. 2025)
Meta-analysis of 24 public datasets	multiple	multiple	positive	2-35%	Number of detected peaks	(Schmid et al. 2021)
NIST human fecal	RPC	Astral	pos	(median) 30%	Number of detected MS1 centroids	(Zamboni 2024)
E.coli, environmental water, human plasma	RPC and HILIC	Orbitrap (ID-X)	pos	(median) 10%	Number of detected centroids in EICs matching masses reported in structural databases	(Giné et al. 2021)
Chemical standards	flow-injection	Orbitrap (ID-X)	neg	(median) 50%	Number of detected standard $[M-H]^-$ adducts	(El Abiead, Rutz, et al. 2025)
Chemical standards	flow-injection	Orbitrap (ID-X)	pos	(median) 67%	Number of detected standard $[M+H]^+$ adducts	(El Abiead, Rutz, et al. 2025)
Chemical standards	flow-injection	Orbitrap (ID-X)	pos	(median) 0%	Number of detected standard $[M+Na]^+$ adducts	(El Abiead, Rutz, et al. 2025)
Chemical standards	HILIC	qToF (Bruker Impact II)	neg	44%	Number of detected standards	(Guo et al. 2021)
Chemical standards	RPC	qToF (Bruker Impact II)	pos	67%	Number of detected standards	(Guo et al. 2021)
Chemical standards	unknown	ToF/qToF (Agilent)	unknown	(mean) ~70%	Number of detected standards	(Giera et al. 2024)

Chemical standards	RPC	Orbitrap (unknown)	pos	63%	Number of detected standards	(Chen et al. 2023)
Chemical standards	flow-injection	Orbitrap (ID-X)	both	(median) 7%	Number of detected MS1 centroids	(Zamboni 2024)
Chemical standards	flow-injection	qToF (SCIEX 7600 ZenoTOF)	both	(median) 34%	Number of detected MS1 centroids	(Zamboni 2024)

Supplementary Information 1

Case Study 1: Bile acids.

We created a library of MS/MS spectra from the GNPS/MassIVE data repository to enable comprehensive annotation of bile acid-related signatures using standard spectral matching tools. Unlike conventional libraries, our goal was to include all observed ion forms, including ISFs that report on bile acids that we could find in the public domain. Using MassQL, we queried 1.2 billion public MS/MS spectra for diagnostic fragment ions characteristic of the bile acid core structure, yielding 21,549 bile acid-related MS/MS signatures. These formed a new community-accessible spectral resource. FDR against GNPS libraries was <1%, suggesting fewer than 21 false positives in the final set. Using ReDU metadata, we linked these spectra to organ sites, health conditions, diet, and antibiotic exposure. We validated 13 predicted polyamine-conjugated bile acids via co-migration and MS/MS matching, and synthesized five more with close spectral matches but differing retention times, indicating possible isomerism. Since then, ~3,700 of those MS/MS spectra of the bile acids library now have MS/MS that match from synthetic standards. Inclusion of ISFs reflects real ion forms observed in biological data and strengthens the use of the library for annotation of bile acids.

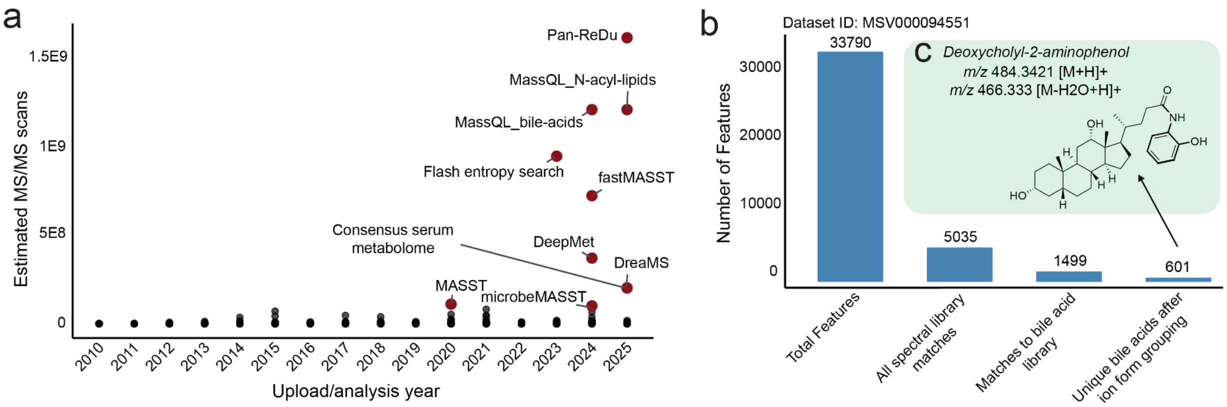

Supplementary Figure 2. Leveraging ISF in repository scale analyses: a) The scale of MS/MS spectra used for analysis in the last 15 years. The figure is generated by estimating 2K spectra per file deposited in GNPS/MassIVE. In addition, studies performing repository scale analyses across GNPS/MassIVE, Metabolights and Metabolomics Workbench have been manually added to highlight the steep increase in the scale of analysis in the last 2 years (Yurekten et al. 2024; Sud et al. 2016; M. Wang et al. 2016; El Abiead, Strobel, et al. 2025; Bushuiev et al. 2025; Mohanty et al. 2024; Mannochio-Russo et al. 2025; Li and Fiehn 2023; Chi, Mitchell, Thapa, et al. 2025; Zuffa et al. 2024; M. Wang et al. 2020; Mongia et al. 2024). b) Calculation of total features captured by mzmine4, spectral library matching to all GNPS libraries and bile acid specific candidate library and subsequent grouping of ISFs and other ions forms to give 601 unique bile acids in a single dataset in GNPS/MassIVE (MSV000094551). **c)** Discovery of

deoxycholyl-2-aminophenol in the same dataset by spectral matching of the candidate library to the ISF of one water loss. The spectral matching was further confirmed by retention time and ion mobility with the synthetic standard.

From the matching against the 21,549 MS/MS library, ISFs were expected and accepted to maximize bile acid detection coverage, since they share diagnostic ions. However, caution is required when applying the library to experimental data. For example, in a study comparing intestinal fluid and fecal samples, 1,499 MS/MS spectra matched entries in the bile acid library(Mohanty et al. 2025). After grouping based on ion forms, these matches corresponded to 601 unique bile acid structures (**Supplementary Figure 2b**) (Mohanty et al. 2024). This underscores the importance of recognizing and consolidating different ion forms to avoid inflating molecular counts – if the goal is to understand the number of detected bile acids.

Ion form determination becomes even more critical when moving from annotation to full structural identification. In one illustrative case, a match to an ISF in the bile acid library led to the discovery of a previously unreported bile amidate: deoxycholyl conjugated with 2-aminophenol. The initial observation came from ISF-based matches in intestinal and fecal samples, which were supported by the presence of bile acid diagnostic fragments. The compound was confirmed via synthesis (**Supplementary Figure 2c**) and validated by MS/MS spectral matching, retention time, and ion mobility analysis against the synthetic standard (Mohanty et al. 2025).

Case Study 2: N-acyl lipids.

Here, ISFs were explicitly excluded. We created MassQL filters for 8,256 theoretical N-acyl lipids based on combinations of 64 amines/amino acids with fatty acids (C2-C30, 0-4 double bonds), and unlike the bile acid library, targeted only candidate $[M+H]^+$ ions. This retrieved 851 N-acyl lipids from public data, 777 of which were not in existing lipid structural databases. A subset (347) was confirmed in data from microbial cultures. For histamine N-acyl lipid conjugates, we confirmed microbial origin via microbial culturing experiments with histamine and co-migrations with synthetic standards. ReDU metadata showed links to diet, colonization, and HIV status. Histamine conjugates correlated with HIV markers; polyamine conjugates with neurocognitive status.

Case Study 3: Drug analog library

We created a reference MS/MS library of drugs and their structural analogs, explicitly including ISFs and other ion forms (Zhao et al. 2024). Because the primary goal of this work is to detect drug exposure, it is essential to annotate not only the molecular ions but also related ion forms such as ISFs, adducts, and isotopes to achieve a comprehensive picture of exposure – as some might only be observed as ISF depending on data acquisition parameters. Using MASST, we expanded the collection via analog searches in GNPS, MetaboLights, and Metabolomics Workbench. Analysis based on peak shape and fragmentation patterns revealed that approximately 33% of the recovered spectra represented ion forms other than the monoisotopic (de)protonated ion: isotopes (5%), other adducts (17%), ISFs (11%).

Together, these examples show how repository-scale analyses can enable new discoveries, and how ISFs may be either included or excluded depending on the specific

analytical context. As increasingly complex questions are posed at the repository level, it becomes clear that determining whether a spectral feature represents an ISF or another ion form will remain critical in many scenarios.

References

Bushuiev, Roman, Anton Bushuiev, Raman Samusevich, Corinna Brungs, Josef Sivic, and Tomáš Pluskal. 2025. “Self-Supervised Learning of Molecular Representations from Millions of Tandem Mass Spectra Using DreaMS.” *Nature Biotechnology*, May, 1–11.

Chen, Lin, Hong Pan, Guohong Zhai, Qi Luo, Yi Li, Chao Fang, and Fuguo Shi. 2023. “Widespread Occurrence of in-Source Fragmentation in the Analysis of Natural Compounds by Liquid Chromatography-Electrospray Ionization Mass Spectrometry.” *Rapid Communications in Mass Spectrometry: RCM* 37 (12): e9519.

Chi, Yuanye, Joshua M. Mitchell, Maheshwor Thapa, Shujian Zheng, Zackary Frohock, Yi Li, Aleksandr Smirnov, Xiuxia Du, and Shuzhao Li. 2025. “Constructing a Consensus Serum Metabolome.” *Biochemistry*. bioRxiv. https://www.biorxiv.org/content/10.1101/2025.05.07.652782v1.

Chi, Yuanye, Joshua M. Mitchell, Shujian Zheng, and Shuzhao Li. 2025. “Systematic Pre-Annotation Explains the ‘Dark Matter’ in LC-MS Metabolomics.” *bioRxivorg*. https://doi.org/10.1101/2025.02.04.636472.

El Abiead, Yasin, Adriano Rutz, Simone Zuffa, Bashar Amer, Shipei Xing, Corinna Brungs, Robin Schmid, et al. 2025. “Discovery of Metabolites Prevails amid in-Source Fragmentation.” *Nature Metabolism* 7 (3): 435–37.

El Abiead, Yasin, Michael Strobel, Thomas Payne, Eoin Fahy, Claire O’Donovan, Shankar Subramamiam, Juan Antonio Vizcaíno, et al. 2025. “Enabling Pan-Repository Reanalysis for Big Data Science of Public Metabolomics Data.” *Nature Communications* 16 (1): 4838.

Giera, Martin, Aries Aisporna, Winnie Uritboonthai, and Gary Siuzdak. 2024. “The Hidden Impact of in-Source Fragmentation in Metabolic and Chemical Mass Spectrometry Data Interpretation.” *Nature Metabolism*, June. https://doi.org/10.1038/s42255-024-01076-x.

Giné, Roger, Jordi Capellades, Josep M. Badia, Dennis Vughs, Michaela Schwaiger-Haber, Theodore Alexandrov, Maria Vinaixa, Andrea M. Brunner, Gary J. Patti, and Oscar Yanes. 2021. “HERMES: A Molecular-Formula-Oriented Method to Target the Metabolome.” *Nature Methods* 18 (11): 1370–76.

Guo, Jian, Sam Shen, Shipei Xing, Huaxu Yu, and Tao Huan. 2021. “ISFrag: De Novo Recognition of in-Source Fragments for Liquid Chromatography-Mass Spectrometry Data.” *Analytical Chemistry* 93 (29): 10243–50.

Li, Yuanyue, and Oliver Fiehn. 2023. “Flash Entropy Search to Query All Mass Spectral Libraries in Real Time.” *Nature Methods* 20 (10): 1475–78.

Lu, Wenyun, Xi Xing, Lin Wang, Li Chen, Sisi Zhang, Melanie R. McReynolds, and Joshua D. Rabinowitz. 2020. “Improved Annotation of Untargeted Metabolomics Data through Buffer Modifications That Shift Adduct Mass and Intensity.” *Analytical Chemistry* 92 (17): 11573–81.

Mahieu, Nathaniel G., and Gary J. Patti. 2017. “Systems-Level Annotation of a Metabolomics Data Set Reduces 25 000 Features to Fewer than 1000 Unique Metabolites.” *Analytical Chemistry* 89 (19): 10397–406.

Mannochio-Russo, Helena, Vincent Charron-Lamoureux, Martijn van Faassen, Santosh Lamichhane, Wilhan D. Gonçalves Nunes, Victoria Deleray, Adriana V. Ayala, et al. 2025. “The Microbiome Diversifies Long- to Short-Chain Fatty Acid-Derived N-Acyl Lipids.” *Cell* 0

(0). https://doi.org/10.1016/j.cell.2025.05.015.
Mohanty, Ipsita, Helena Mannochio-Russo, Joshua V. Schweer, Yasin El Abiead, Wout Bittremieux, Shipei Xing, Robin Schmid, et al. 2024. “The Underappreciated Diversity of Bile Acid Modifications.” *Cell* 187 (7): 1801–18.e20.
Mohanty, Ipsita, Shipei Xing, Vanessa Castillo, Julius Agongo, Abubaker Patan, Yasin El Abiead, Helena Mannochio-Russo, et al. 2025. “MS/MS Mass Spectrometry Filtering Tree for Bile Acid Isomer Annotation.” *bioRxiv*. https://doi.org/10.1101/2025.03.04.641505.
Mongia, Mihir, Tyler M. Yasaka, Yudong Liu, Mustafa Guler, Liang Lu, Aditya Bhagwat, Bahar Behsaz, Mingxun Wang, Pieter C. Dorrestein, and Hosein Mohimani. 2024. “Fast Mass Spectrometry Search and Clustering of Untargeted Metabolomics Data.” *Nature Biotechnology* 42 (11): 1672–77.
Schmid, Robin, Daniel Petras, Louis-Félix Nothias, Mingxun Wang, Allegra T. Aron, Annika Jagels, Hiroshi Tsugawa, et al. 2021. “Ion Identity Molecular Networking for Mass Spectrometry-Based Metabolomics in the GNPS Environment.” *Nature Communications* 12 (1): 3832.
Sud, Manish, Eoin Fahy, Dawn Cotter, Kenan Azam, Ilango Vadivelu, Charles Burant, Arthur Edison, et al. 2016. “Metabolomics Workbench: An International Repository for Metabolomics Data and Metadata, Metabolite Standards, Protocols, Tutorials and Training, and Analysis Tools.” *Nucleic Acids Research* 44 (D1): D463–70.
Wang, Lin, Xi Xing, Li Chen, Lifeng Yang, Xiaoyang Su, Herschel Rabitz, Wenyun Lu, and Joshua D. Rabinowitz. 2019. “Peak Annotation and Verification Engine for Untargeted LC-MS Metabolomics.” *Analytical Chemistry* 91 (3): 1838–46.
Wang, Mingxun, Jeremy J. Carver, Vanessa V. Phelan, Laura M. Sanchez, Neha Garg, Yao Peng, Don Duy Nguyen, et al. 2016. “Sharing and Community Curation of Mass Spectrometry Data with Global Natural Products Social Molecular Networking.” *Nature Biotechnology* 34 (8): 828–37.
Wang, Mingxun, Alan K. Jarmusch, Fernando Vargas, Alexander A. Aksenov, Julia M. Gauglitz, Kelly Weldon, Daniel Petras, et al. 2020. “Mass Spectrometry Searches Using MASST.” *Nature Biotechnology* 38 (1): 23–26.
Yurekten, Ozgur, Thomas Payne, Noemi Tejera, Felix Xavier Amaladoss, Callum Martin, Mark Williams, and Claire O’Donovan. 2024. “MetaboLights: Open Data Repository for Metabolomics.” *Nucleic Acids Research* 52 (D1): D640–46.
Zamboni, Nicola. 2024. “70% in-Source Fragments? The Data Tells a Different Story!” Yet Another Metabolomics Club. October 16, 2024. https://metabolomics.blog/2024/10/isf/.
Zhao, Haoqi Nina, Kine Eide Kvitne, Corinna Brungs, Siddharth Mohan, Vincent Charron-Lamoureux, Wout Bittremieux, Runbang Tang, et al. 2024. “Empirically Establishing Drug Exposure Records Directly from Untargeted Metabolomics Data.” *Bioinformatics*. bioRxiv. https://www.biorxiv.org/content/10.1101/2024.10.07.617109v1.
Zuffa, Simone, Robin Schmid, Anelize Bauermeister, Paulo Wender P Gomes, Andres M. Caraballo-Rodriguez, Yasin El Abiead, Allegra T. Aron, et al. 2024. “microbeMASST: A Taxonomically Informed Mass Spectrometry Search Tool for Microbial Metabolomics Data.” *Nature Microbiology* 9 (2): 336–45.
